# Pseudodynamic analysis of heart tube formation in the mouse reveals strong regional variability and early left–right asymmetry

**DOI:** 10.1038/s44161-022-00065-1

**Published:** 2022-05-16

**Authors:** Isaac Esteban, Patrick Schmidt, Audrey Desgrange, Morena Raiola, Susana Temiño, Sigolène M. Meilhac, Leif Kobbelt, Miguel Torres

**Affiliations:** 1 Cardiovascular Regeneration Program, Centro Nacional de Investigaciones Cardiovasculares (CNIC), Madrid, Spain; 2 Universidad Politécnica de Madrid, Madrid, Spain; 3 Visual Computing Institute, RWTH Aachen University, Aachen, Germany; 4 Unit of Heart Morphogenesis, Université de Paris, Imagine - Institut Pasteur, INSERM UMR1163, Paris, France

**Keywords:** Heart development, Developmental biology

## Abstract

Understanding organ morphogenesis requires a precise geometrical description of the tissues involved in the process. The high morphological variability in mammalian embryos hinders the quantitative analysis of organogenesis. In particular, the study of early heart development in mammals remains a challenging problem due to imaging limitations and complexity. Here, we provide a complete morphological description of mammalian heart tube formation based on detailed imaging of a temporally dense collection of mouse embryonic hearts. We develop strategies for morphometric staging and quantification of local morphological variations between specimens. We identify hot spots of regionalized variability and identify *Nodal*-controlled left–right asymmetry of the inflow tracts as the earliest signs of organ left–right asymmetry in the mammalian embryo. Finally, we generate a three-dimensional+t digital model that allows co-representation of data from different sources and provides a framework for the computer modeling of heart tube formation

## Abstract (web summary)

Using high-resolution confocal images and computational surface mapping, Esteban et al. provide a detailed pseudodynamic atlas of early heart tube development (E7.5–E8.5), develop a morphometric staging system based on landmark curves and distances in the surface of the tissues and identify parameters that can be used for precise embryo staging across different labs. This morphometric analysis reveals early signs of left–right asymmetry, before the cardiac looping stage, which is regulated by the Nodal signaling pathway.

## Main

The mammalian embryo is highly regulative, which implies it can consistently develop stereotyped organs despite initial morphological heterogeneity. Heart tube formation involves complex morphogenetic movements and differentiation patterns, through which form and function are acquired simultaneously^1^. Understanding heart tube morphogenesis would greatly benefit from having access to a spatiotemporal representation of the morphology of the tissues involved in the process. Previous attempts to understand the three-dimensional+time (3D+t) complexity of primitive heart tube formation in amniotes have been based on the segmentation and 3D reconstruction of specimens from tissue sections^2–5^. This approach has allowed for the reconstruction of a few discrete stages capturing this process but is limited by a demanding methodology and the distortions of section alignment. Additional efforts have included high-resolution episcopic microscopy and live analysis of the developing chicken or mouse heart; however, this was applied to stages beyond primitive heart tube formation and focused on specific processes of cardiogenesis^6–9^ or was limited by incomplete imaging of the area of interest and low image resolution^10,11^.

Cardiac development starts with the deployment of cardiac mesoderm early in gastrulation^12^. Soon after colonizing the antero–lateral region of the embryo, the cardiac mesoderm at the anterior lateral plate undergoes a mesenchymal-to-epithelial transition to produce two layers that enclose the celomic cavity. This results in two independent epithelial sheets: the splanchnic mesoderm, which shares basement membrane with the endoderm, and the somatic mesoderm, which shares basement membrane with the ectoderm. The two mesodermal layers meet medially with the head paraxial mesoderm and laterally at the embryonic–extraembryonic border, forming a closed and continuous 3D surface encasing the anterior celomic cavity (future pericardial cavity).

During embryonic days 7.5–8.5 (E7.5–E8.5) in the mouse, the splanchnic and somatic mesoderm layers progressively move apart from each other, forming the pericardial cavity. This occurs in parallel to embryonic folding and foregut pocket formation, which brings the foregut endoderm inside the embryo. This causes the pericardial cavity to rotate inwards around the left–right axis, driven by the rostral folding of the embryo, while the endoderm invaginates toward the anterior. Following foregut pocket formation, the pericardial cavity is limited at its cranial side by the ectodermal plate and the head mesoderm. Caudally, the cavity limits with the foregut diverticulum in its medial aspect, while the posterior–lateral limits of the cavity extend around the foregut invagination, ending at the somatic and splanchnic mesoderm junction.

The splanchnic mesoderm of the forming pericardial cavity contains the cardiac mesoderm that gives rise to the heart tube. Within the cardiac mesoderm, there are two distinct regions: the first heart field (FHF), which differentiates rapidly to the cardiomyocyte fate, and the second heart field (SHF), which remains highly proliferative and undifferentiated until its precursors are progressively incorporated to the heart at later stages. In the early embryo, the FHF extends anteriorlaterally, closer to the embryonic–extraembryonic boundary, while the SHF occupies the posterior–medial regions adjacent to the FHF. The initially relatively flat cardiac crescent (CC) starts very active morphogenesis around E7.5, growing and acquiring 3D complexity by folding on itself, detaching from the endoderm and coalescing medially to form the primitive tube, with the venous-to-arterial pole axis in a caudal-to-cranial orientation.

In this work, we aimed to obtain a complete description of tissue anatomy during primitive heart tube morphogenesis in the mouse embryo. To this end, we acquired a collection of high-resolution confocal images from whole specimens at high temporal density and used the images to generate a continuous 3D+t description of heart tube morphogenesis. Given that heart tube morphogenesis cannot be uncoupled from the morphological evolution of the surrounding tissues, our model incorporates all pericardial cavity mesoderm, endoderm and the endothelium. Besides obtaining a detailed 3D+t atlas of heart tube formation and its associated tissues, we applied statistical methods to study regional variability and left–right patterning during heart tube formation. The model and knowledge generated provide a strong basis for understanding heart morphogenesis, its variability and its congenital alterations.

## Results

### High-resolution spatiotemporal study of the developing heart

**Fig. 1:**
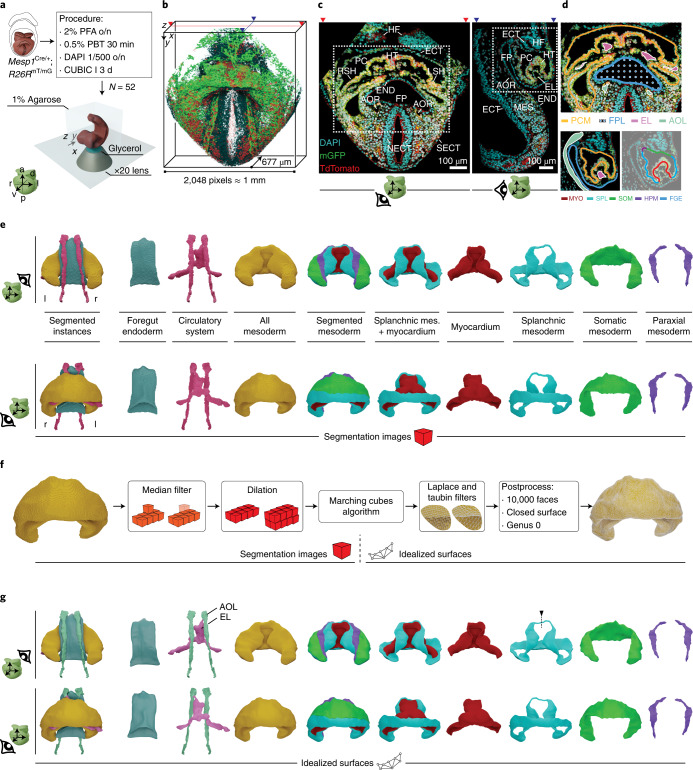
Segmentation of high-resolution confocal images reveals precise and detailed morphological representations for understanding heart development. **a**, Schematic representation of the workflow for image acquisition. The icon below shows the axes of the system of reference; o/n, overnight; PFA, paraformaldehyde; PBT, phosphate buffer saline with 0.5% triton X-100. **b**, Confocal raw image reconstruction showing the dimensions of the acquired volumes. Mesodermal tissues show the cytoplasm in red and cell membranes in green; the rest of the tissues show membranes in red. Cell nuclei appear in cyan. Red and blue arrowheads indicate the optical sections shown in **c**. **c**, Frontal (left) and sagittal (right) optical sections. Dotted-line boxes highlight the cardiac region. **d**, Top, frontal optical section at the level of the cardiac inflow tract (IFT) region. The orange line shows the segmented mesoderm of the anterior celomic cavity, including the differentiating myocardium, and the foregut endoderm is represented in blue. The foregut pocket lumen is represented using a blue-dot grid. The endocardial lumen is shown in pink, and the aortic lumen is shown in green. Bottom left, lateral sagittal optical section at the level of one of the aortae. Bottom right, the same optical section showing the subtypes of mesoderm of the pericardial cavity. Myocardium is in red, splanchnic mesoderm is in blue, somatic mesoderm is in green, and head paraxial mesoderm is in purple. **e**, Dorsal (top) and ventral (bottom) 3D representation of the segmented tissues; mes. mesoderm. **f**, Pipeline for transforming the segmentation images into smooth, closed meshes. **g**, Dorsal (top) and ventral (bottom) views of the resulting meshes. The black dotted arrow represents the cut trajectory to achieve genus 0 topology in the splanchnic shapes (Methods). Abbreviations: a, anterior; p, posterior; r, right; l, left; v, ventral; d, dorsal; HF, head folds; HT, heart tube; ECT, ectoderm; PC, pericardial cavity; RSH, right sinus horn; LSH, left sinus horn; END, endoderm; FP, foregut pocket; AOR, aorta; SECT, surface ectoderm; NECT, neuroectoderm; EL, endocardial lumen; MES, mesoderm; PCM, pericardial cavity mesoderm; FPL, foregut pocket lumen; FGE, foregut endoderm; AOL, aortic lumen; MYO, myocardium; SPL, splanchnic mesoderm; SOM, somatic mesoderm; HPM, head paraxial mesoderm.

To generate a high-fidelity 3D+t atlas of early mouse heart development, we imaged 52 fixed and cleared^13^ embryos in cardiac developmental stages ranging from the early CC until heart looping. The collection represents nominal ages from approximately E7.75 to E8.5 (18 h). The estimated approximate temporal density is therefore one specimen every 20 min. The imaged embryos carried *Mesp1*^*Cre*^ and *R26R*^*mTmG*^ and *R26R*^*Tomato*^ alleles so that all mesoderm in the head and cardiogenic area was labeled with membrane green fluorescent protein (mGFP) and cytoplasmic Tomato, while the rest of the tissues were labeled only with membrane Tomato. We imaged volumes of ~1 mm in the *x* and *y* dimensions and down to a depth of 677 µm, so that the whole spatial domain of the cardiogenic region and associated tissues was acquired. We used an *x*/*y* pixel size varying from 0.38 µm to 0.49 µm and a *z* step varying from 0.49 µm to 2.0 µm, depending on the specimen. The varying ranges in resolution were adapted for optimization of acquisition time and minimization of photobleaching with the larger specimens. This strategy provided highly detailed orthogonal optical sections at resolutions beyond the need for 3D segmentation at tissue level (Fig. 1c).

We then semiautomatically segmented tissues of interest in each two-dimensional (2D) confocal slice (Methods). The segmented tissues included the mesoderm of the anterior celomic cavity, discriminating both layers of the lateral plate mesoderm, splanchnic and somatic, up to their fusion with the head paraxial mesoderm (Fig. 1d). Within the splanchnic domain, we used morphological and topological criteria to discriminate the differentiated myocardium from the rest of the splanchnic mesoderm. The cells that were detached from the endoderm and encased, or overlayed, endocardial cells were classified as differentiating cardiomyocytes (Fig. 1d). In addition, we segmented the foregut endoderm, the endocardial lumen and the forming aortae (Fig. 1d and Methods). The 2D segmentations of the different tissues were then used to reconstruct primary 3D images (Fig. 1e), which were subsequently smoothened and transformed into meshes (Fig. 1f and Methods). The meshes constitute closed surfaces describing the interface of each segmented tissue with the surrounding tissues or cavities. In this way, we generated 3D models for the 52 specimens of the collection (an example is shown in Fig. 1g). These 3D models are available for download (see Data availability) and can be visualized using, for example, the open-source application ParaView (www.paraview.org).

**Fig. 2:**
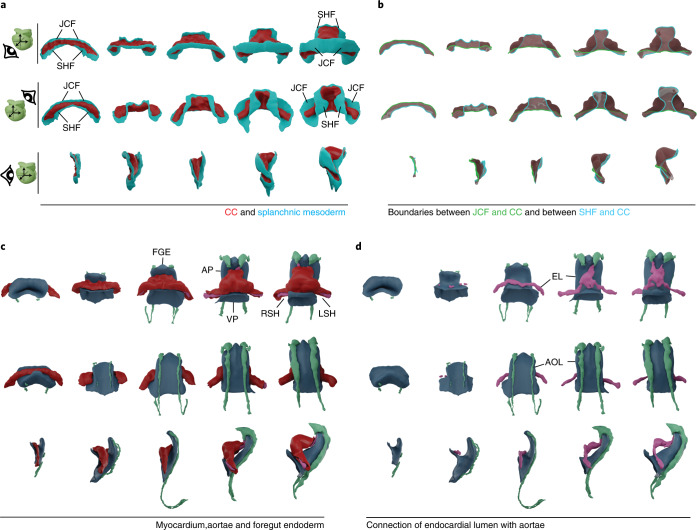
Observation of qualitative morphometric aspects of heart tube morphogenesis. **a**, Ventral, dorsal and lateral views of five specimens that are subjectively ordered according to developmental time. Splanchnic mesoderm is represented in blue, and differentiated myocardium is represented in red. The dorsal view shows the progressive dorsal closure of the heart tube and the medial expansion of the SHF. **b**, Limits of the myocardial differentiated domain depicted on the edges of the myocardium midsurface. The green line represents the boundary between the juxta-cardiac field and the myocardium. The blue line represents the boundary between the myocardium and the SHF. **c**, Morphology of the myocardium together with the foregut endoderm and the circulatory system lumen. The different lengths of the aortae seen in the three older specimens are due to variable imaging depth. **d**, Gradual development of the circulatory system in parallel to the foregut endoderm invagination. The ventral view shows the progressive formation of the endocardial lumen, and the lateral view shows the progressive formation of the aortic lumen along the dorsal surface of the foregut endoderm. Abbreviations: JCF, juxta-cardiac field; FGE, foregut endoderm; AP, arterial pole; VP, venous pole; EL, endocardial lumen; AOL, aortic lumen; LSH, left sinus horn; RSH, right sinus horn.

Preliminary study of five selected specimens in obvious temporal sequence (Fig. 2a,b) showed how the splanchnic mesoderm folds to form the CC. The early CC extends bilaterally and subdivides the undifferentiated splanchnic mesoderm into medial and distal domains. The medial domain contains the SHF precursors, while the distal domain (recently named juxta-cardiac field) contains precursors of the epicardium and the septum transversum and bears myocardial differentiation capacity^14^. As the CC undergoes morphogenesis to generate the cardiac tube, both the juxta-cardiac field and its border with the forming heart tube remain morphologically rather stable (Fig. 2a,b), whereas the medial splanchnic mesoderm (containing the SHF) and its border with the forming heart tube undergo a drastic deformation that is essential for the generation of the outflow tract (OFT), allowing the dorsal closure, which transforms the CC into the primitive heart tube (Fig. 2a,b). This transformation is concomitant with the development of the endocardial cavity, the aortae and their connection through the first branchial arch arteries (Fig. 2c,d).

### Morphometric staging of heart tube formation

**Fig. 3:**
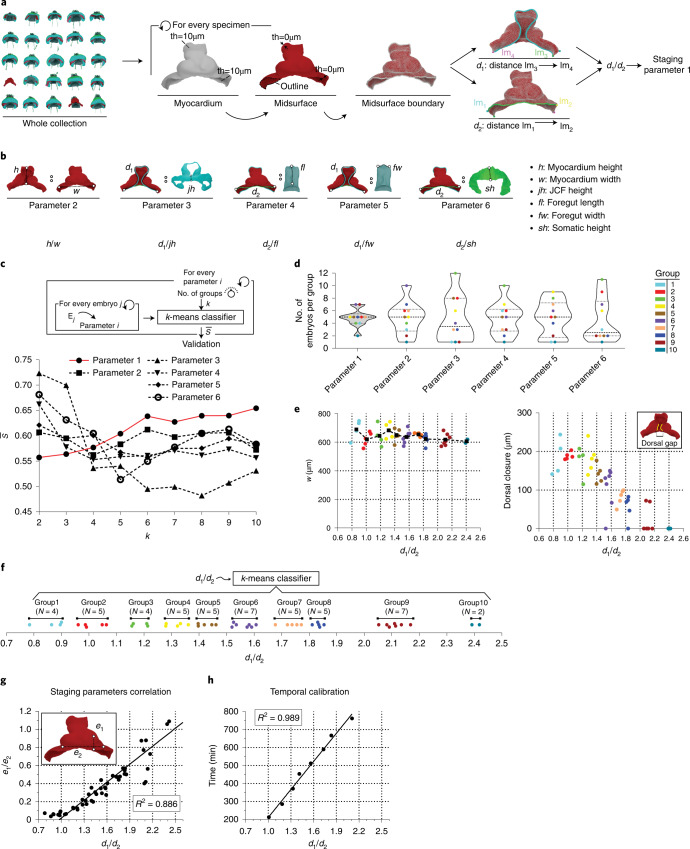
Establishment of a morphometric staging system. **a**, Schematic representation for the calculation of staging parameter 1. lm, landmark; th, thickness. **b**, Schematic representation of five additional staging parameters. **c**, Top, workflow for evaluating staging parameters by means of the average silhouette coefficient ($\bar s$). Bottom, graph showing the evolution of $\bar s$ as a function of the number of groups for every staging parameter. **d**, Violin plots showing the distribution of specimens in each cluster (*N* = 10 clusters) for the six selected staging parameters. **e**, Scatter plots showing myocardium width (left) and dorsal gap width (right) as a function of *d1*/*d2*. **f**, Plot showing the final classification of the collection in 10 groups according to *d1*/*d2*. **g**, Correlation between *d1*/*d2* and the staging parameter applied to live-imaging data (*e1*/*e2*). **h**, Temporal calibration of the progression of the *d1*/*d2* values. Source data

**Extended Data Fig. 1:**
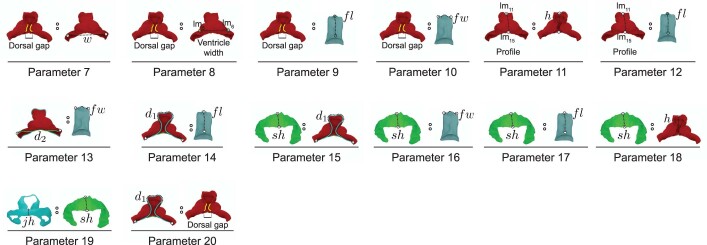
Additional parameters explored for staging. Schematic representation of additional parameters explored for the staging system.

We next aimed the temporal ordering of the specimen collection. The examination of the collection of datasets reflected high morphological heterogeneity of the developing heart within groups of embryos at an apparently similar developmental stage, as judged by somite number, head folds and foregut pocket shapes. The morphological heterogeneity affects both general conformation features, such as proportions, degree of dorsal closure or degree of looping, and local morphological aspects, such as non-stereotyped presence of bulges of different sizes and distribution. To tackle the problem of staging despite this variability, we implemented different morphometric strategies. We first obtained midsurfaces^15^ for each of the relevant tissues, consisting of a zero-thickness surface resulting from flattening the original surfaces (Fig. 3a), and defined various landmark distances and curves on such surfaces (Fig. 3b and Extended Data Fig. 1). We next studied the proportions between the lengths of pairs of the reference curves/distances (Fig. 3b) and focused on proportions expected to vary continuously during the studied period (See example in Fig. 3a).

**Extended Data Fig. 2:**
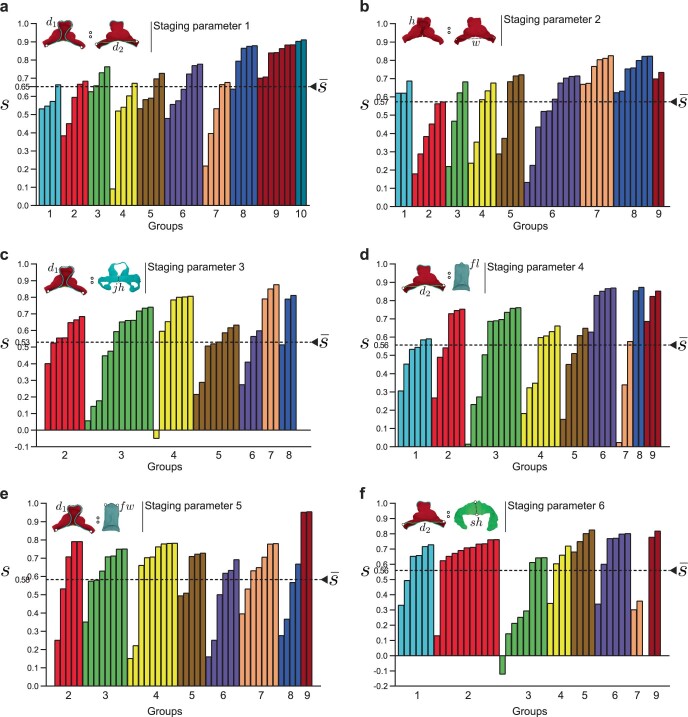
Silhouette coefficient plot for the different staging parameters explored, classified using k-means and number of groups equal to 10. **a**-**f**, Bar graphs showing the silhouette coefficient value (*s*) for each embryo in the collection classified into 10 groups, according to the different staging parameters. The graphs also show the average value ($\bar s$), which is maximal for parameter 1. Graphs are color-coded according to the table shown in Fig. 3D. Schemes above the graphs show a representation of staging parameter used. Source data

**Extended Data Fig. 3:**
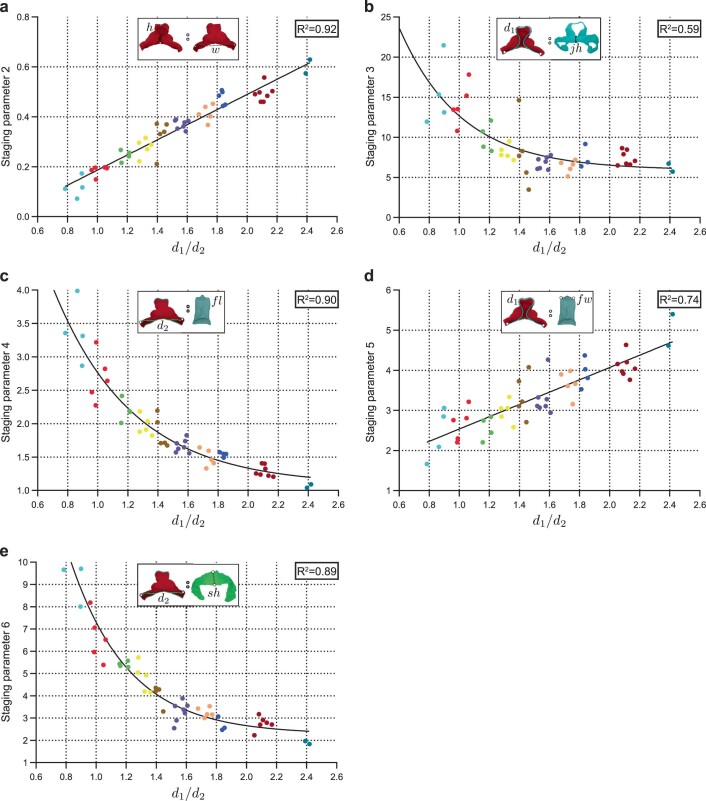
Correlations between staging parameters unveil tight relationships between the evolution of different morphometric aspects during heart development. **a**, **b**, **c**, **d**, **e**, Plots show the correlation of staging parameters 2-6 versus staging parameter 1, *d1/d2*. Source data

Given that the cardiogenic area evolves from a crescent extending from left to right to a tube extending from cranial to caudal, a general trend during the formation of the heart tube is the increase of the height (cranio–caudal size) to width (left–right size) ratio of the tissues involved. We therefore calculated the proportions between different parameters related to the height-to-width ratio variation (Fig. 3a,b, parameters 1 to 6). We then applied the *k*-means^16^ clustering algorithm for the determination of groups based on differences in the defined parameters and calculated the average silhouette coefficient ($\bar s$; Extended Data Fig. 2), which measures the quality of classifications^17^. As we increased the number of groups (*k*) in the *k*-means classification, the best $\bar s$ was obtained for the *d1*/*d2* proportion (Fig. 3c), *d1* being the length of the border between the myocardium and SHF and *d2* being the length of the border between the myocardium and juxta-cardiac field (Fig. 3a). The maximum $\bar s$ was obtained for a *k*-means classification into 10 groups. We therefore concluded that *d1*/*d2* was the best single parameter for classifying the collection and established these 10 groups as a reference for study (Fig. 3f). While the establishment of groups is useful to understand the morphological evolution of the heart, the *d1*/*d2* parameter is a continuous variable and allows the allocation of single specimens along the heart tube developmental trajectory, independently of grouping. Study of the correlation between *d1*/*d2* and the rest of the parameters provided a view of how good the different classification methods are with respect to *d1*/*d2* (Extended Data Fig. 1). The best correlate to *d1*/*d2* was the height-to-width ratio, which showed a high linear correlation (Extended Data Fig. 3a). By contrast, other parameters either showed a poor correlation or did not show a linear correlation, which resulted in poor classification of the older specimens (Extended Data Fig. 3). The *d1*/*d2* proportion also provided the most homogeneous distribution of specimens among the 10 groups (Fig. 3d).

The geometries of all the tissues segmented for every specimen classified by stage are shown in Supplementary Figs. 1–8. Groups 1 to 4 represent different stages of CC development, while groups 5 to 8 can be assigned to linear heart tube stages and groups 9 and 10 to heart looping stages. Interestingly, the absolute width of the forming cardiac tube, which approximately coincides with the width of the pericardial cavity, not only does not increase during development but shows a mild trend to reduction as development progresses (Fig. 3e). This aspect indicates that, despite overall embryo growth, the lateral expansion of the pericardial cavity seems restricted. By contrast, the dorsal–ventral and cranio–caudal dimensions of the pericardial cavity and all associated tissues, including the forming heart tube, strongly increase during this period. This aspect correlates well with the extensive deformation that the curve *d1* undergoes, which is associated with myocardial and splanchnic mesoderm remodeling, in contrast with *d2*, which mostly remains stable.

**Extended Data Fig. 4:**
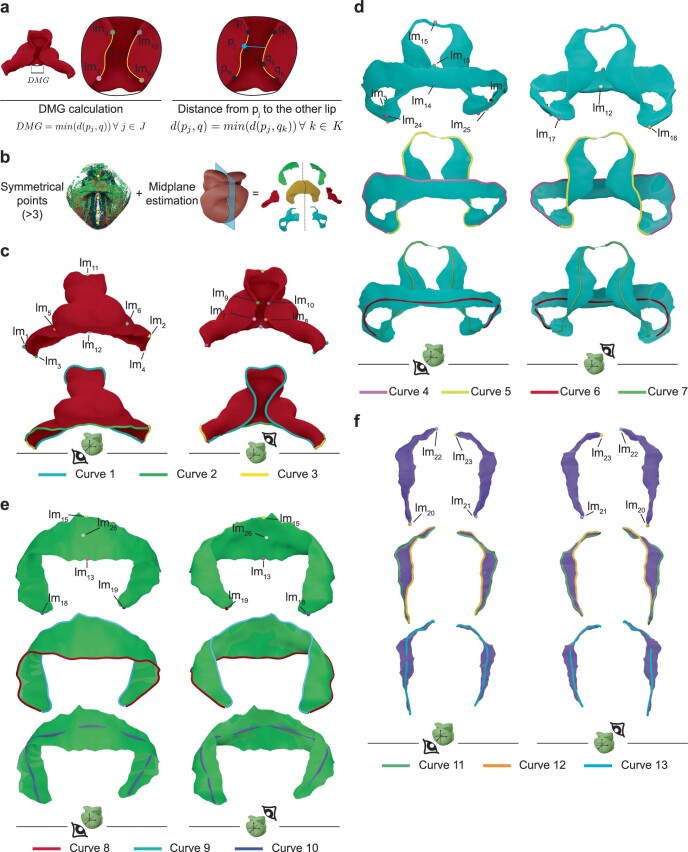
Measurement of dorsal closure and definition of landmarks and curves for surface map computation and shape registration. **a**, Schematic representation of the procedure for estimating the dorsal mesocardial gap (DMG). **b**, This panel shows the process for estimating the mid-sagittal plane of the embryo. The right part shows the tissues of the pericardial cavity cut in half trough the plane. A minimum of 3 mid-sagittal points determined in the raw images were used to calculate the mid-sagittal plane. **c**, The panel shows the basic set of landmarks defined for the myocardium. lm1, lm2, lm3 and lm4 were set at the lateral ends of the IFTs. lm5 and lm6 are located at the point where the IFTs join the ventricle. lm7, lm8, lm9 and lm10 are located at the ends of the closing dorsal lips. lm11 is located at the cranial-most point of the intersection between the mid-sagittal plane and the OFT. lm12 is located at the intersection between the mid-sagittal plane and the line between the myocardium and the juxta-cardiac field. The curves extracted from the edge of the mid-surface are: curve1, from lm3 to lm4; curve2, from lm1 to lm2; curve3, from lm1 to lm3 (left) and lm2 to lm4 (right). **d**, The panel shows the set of landmarks defined in the splanchnic mesoderm. lm3, lm4 and lm12 are the nearest vertex to those in the myocardial landmarks. lm13 is defined at the middle position of the intersection between the splanchnic and the somatic mesoderm. lm15 is at the cranial-most point of the intersection between the mid-sagittal plane and the splanchnic mesoderm. lm14 is obtained by calculating the midpoint of the geodesic curve that joins lm12 and lm13. Lm16 and lm17 are located at the bilateral most caudal points of the splanchnic. lm24 and lm25 are at the midpoint of the geodesic curve that joins lm16 with lm4 and lm17 with lm3, respectively. The curves extracted from the edge of the mid-surface are: curve4, at the border between the splanchnic and somatic mesoderm; curve5, a bilateral curve joining lm24, lm15 and lm25; curve6, joins lm24 and lm25 running equidistant between curve2 and curve4; curve7 is similar to curve5, but running equidistant between curve1 and curve5. **e**, The panel shows the basic set of landmarks defined in the somatic mesoderm. lm13 and lm15 are located at the midpoint of the outer edge of the somatic mesoderm. lm26 is at the midpoint of the geodesic curve that joins lm13 and lm15. lm18 and lm19 are the most caudal bilateral points of the somatic mesoderm. The curves extracted from the edge of the mid-surface are: curve8, at the border between the somatic and the splanchnic mesoderm; curve9, from lm18 to lm19, on the border between somatic and the paraxial mesoderm; curve10, starts at lm18 and ends at lm19 running equidistant between curve8 and curve9. **f**, The panel shows the basic set of landmarks defined in the head paraxial mesoderm. lm20 and lm21 are at the bilateral caudal-most points of the paraxial mesoderm. lm22 and lm23 are defined at the bilateral cranial-most points of the paraxial domain. The curves extracted from the edge of the mid-surface are: curve11, running between the paraxial and somatic mesoderm; curve12, running between the paraxial and the splanchnic mesoderm; curve13, running equidistant between curve11 and curve12.

We also studied the occurrence of developmental events, such as dorsal closure and looping, which are known to progress continuously in live-imaged single specimens^10,11^. Heart tube rotation and looping, as described in ref. ^11^, is appreciable in all specimens of group 10 and most specimens of group 9 (except E42 and E46), without signs of looping in specimens of other groups (Supplementary Figs. 3–6). We also measured the dorsal mesocardial gap as a read out of dorsal closure progression (Extended Data Fig. 4a). This gap is initially wide open in the CC and progressively narrows until disappearing concomitantly with dorsal mesocardium establishment and linear heart tube formation. While dorsal closure appeared predominantly associated with looping and, therefore, was seen in all specimens in group 10+ and most specimens in group 9, we also found some hearts already closed in group 8 (E41) and group 6 (E29), while some specimens at stage 9 were clearly advanced in looping but quite delayed in dorsal closure (E44 and E48; Supplementary Figs. 3–6). In agreement with these observations, dorsal gap correlation with *d1*/*d2* was quite noisy (Fig. 3e), indicating that the degree of dorsal closure is not a reliable parameter for staging.

Finally, we wanted to obtain a real-time calibration of the staging system generated. For this, we analyzed 3D+t datasets previously generated in our lab that capture heart tube formation in vivo^10^. The limitations of the live-imaging setup did not allow us to measure *d1*/*d2* or *h*/*w*; however, we established that the proportion of the ventral protrusion of the primitive chamber to its lateral extension was measurable in live imaging and correlated reasonably well with *d1*/*d2* (Fig. 3g). Using this approach, we correlated the live-imaging timing with *d1*/*d2* (Fig. 3h). This study showed that the available live-imaging data corresponded to the progression from group 1 to group 9, that progression between these stage groups spanned 12 h 42 m and that *d1*/*d2* and real developmental time showed a linear relationship during this period. These results show that during the live-imaging observation period, and with the caveat of the ex vivo approach, *d1*/*d2* represents a valid surrogate of developmental time.

### Three-dimensional atlas of heart tube formation with local shape variability

**Fig. 4:**
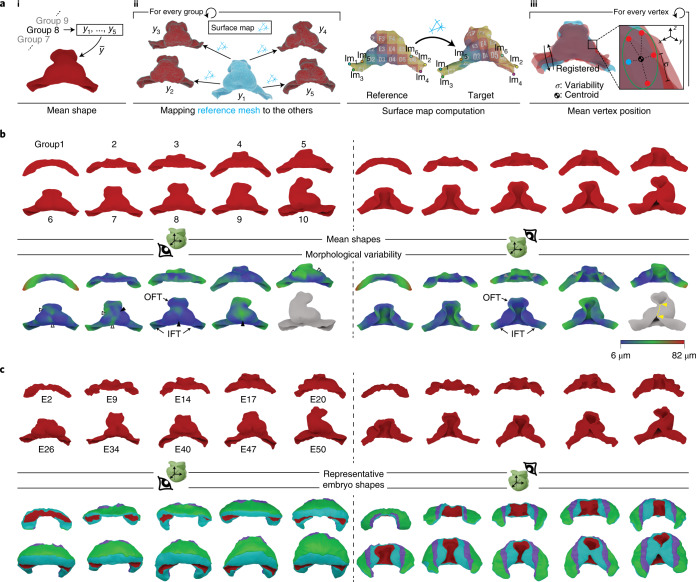
MSG and morphological variability. **a**, Illustration of the concept of mean shape for staging group 8 (i) and workflow for obtaining a mean shape and local variability estimation for each staging group (ii). In ii, the left shows the process of surface map computation. The blue shape represents the mesh of the reference specimen of the group. The arrows represent the surface mapping from the reference specimen mesh to all others in the staging group. The right shows the working principle of the surface map computation. The landmarks (lm) identify corresponding locations in the reference and target mesh. Shapes of the group after rigid registration to the reference mesh are also shown (iii). Every vertex in the reference mesh (blue dot) has a corresponding point on the other shapes (red dots). **b**, Calculated myocardium mean shapes for every staging group. The two top rows show the mean shapes from ventral (left) and dorsal (right) views. The two bottom rows show the same mean shapes color coded according to the morphological variability calculated for each vertex. The shape in gray indicates that the low number of specimens at this stage precludes variability calculation. The yellow dotted line and arrows represent the cut trajectory to achieve genus 0 topology in dorsally closed hearts (Methods). Arrowheads indicate the regions of higher variability in medial ventricular regions (solid) and lateral ventricular regions (open). **c**, Representative specimens of each group. Top, ventral and dorsal view of the myocardium shape. Bottom, ventral and dorsal views of the pericardial tissues of the representative embryos.

Given the variability found between specimens in the same staging groups (Supplementary Figs. 1–8), we next aimed to quantify and spatially map the shape variability of the differentiated myocardium within each of the 10 groups established and to define a consensus geometry for each group (Fig. 4a(i)). For each group, we chose a seed specimen whose *d1*/*d2* was closest to the average *d1*/*d2* of the group (Fig. 4a(ii), *y*_1_ specimen). We then used a described methodology^18^ that uses a set of landmark points to establish a vertex-to-vertex correspondence between all shapes of a group and the seed specimen (Fig. 4a(ii), Extended Data Fig. 4 and Methods). This generated a cluster of corresponding vertices for each position in the mesh. Next, we found the average position of each cluster of corresponding vertices and constructed a mean shape for each group (MSG) composed of the average positions (Fig. 4a(iii)). This generated a collection of 10 MSGs that represents the average evolution of myocardial shape during the formation of the primitive heart tube and initiation of looping (Fig. 4b).

We then computed the per-vertex variability among the specimens of each staging group and plotted it on the MSG collection (Fig. 4a(iii)). In this way, we were able to identify hot spots of variability within each of the 10 different groups (Fig. 4b). In most groups, we found that high variability appeared in the OFTs, inflow tracts (IFTs) and dorsal lips of the myocardium. During primitive ventricular chamber formation, we found that variability concentrated first at the bilateral bulges that start to form the chamber and later at the midline, possibly in relation to the variability in the degree of merging of the two initially bilateral bulges into one. Finally, in group 9, high variability was observed in all structures involved in the looping process (Fig. 4b). Variability was not measured in group 10, given that this group is composed of only two specimens. This collection of MSGs provides information on the standard shapes of different stages of primitive heart tube formation and the local variability of the process.

We also wanted to identify the specimen whose myocardium best represents its stage group, as this would give us access to representative geometries of the other tissues as well. For this, we identified the medoid shape for each group, that is, the geometry with the smallest accumulated difference with respect to all others in the group (Methods). In this way, we identified a concrete specimen as representative shape for each group (RSG; Fig. 4c).

### Early left–right asymmetry of the cardiac IFTs

**Fig. 5:**
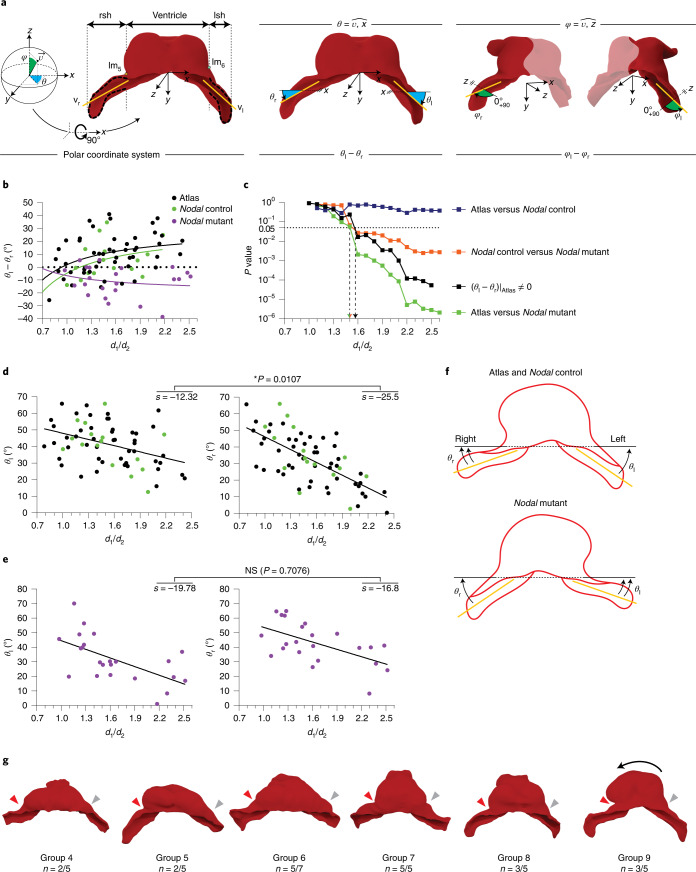
Onset of left–right morphological asymmetry before heart looping. **a**, Schemes show the calculated principal directions of the IFT (orange lines) and the polar coordinates system with *θ* and *φ* angles. rsh, right sinus horn; lsh, left sinus horn; v_r_, vectorial direction of rsh; v_l_, vectorial direction of lsh. **b**, Plot showing the evolution of the *θ* angles depending on the *d1*/*d2* value. Curves represent Padé approximations of every group of specimens. **c**, Evolution of *P* values as a function of *d1*/*d2* for the deviation of the *θ* angle from left–right symmetry and for the comparisons between experimental groups, as indicated. A mixed linear model was applied to estimate the asymmetry between both sides at different stages (fixed effects) adjusting by the angle with two-sided *P* value calculation and applying Sidak’s correction for multiple tests. **d**, Separated values of *θ*_l_ *and θ*_r_ for the two groups of control specimens. Analysis of covariance has been applied to test whether slopes are significantly different calculating two-tailed *P* values; NS, not significant. **e**, Separated values of *θ*_l_ *and θ*_r_ for the *Nodal*-mutant specimens. The statistical analysis was the same as in **d**. **f**, Schematic representation of the different evolution of *θ*_l_ *and θ*_r_ angles in wild-type and *Nodal*-mutant embryos. **g**, Specific wild-type hearts from different groups, highlighting the acute angle between the right IFT insertion and the ventricle (red arrow) and the smooth transition on the opposite side (gray arrow), often accompanied by a small bulge; *n*, number of specimens showing this feature over the total number of specimens in each staging group. The black arrow illustrates the direction of looping. Source data

**Extended Data Fig. 5:**
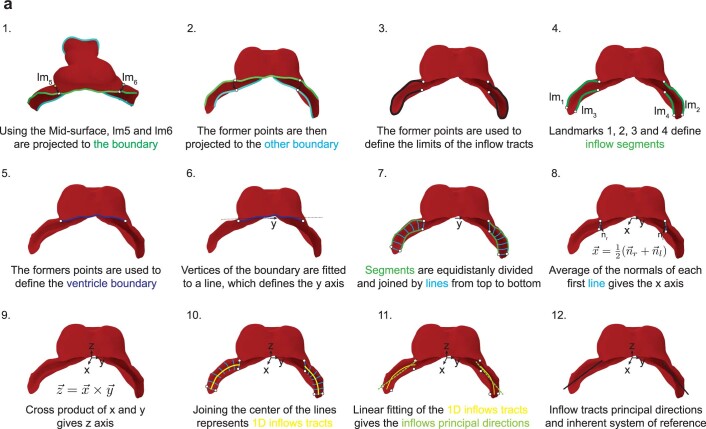
Proposed algorithm for systematic calculation of a myocardium inherent system of reference and calculation of IFT insertion direction. Steps followed for determining the principal direction of the IFTs and a system of reference based on morphology.

**Extended Data Fig. 6:**
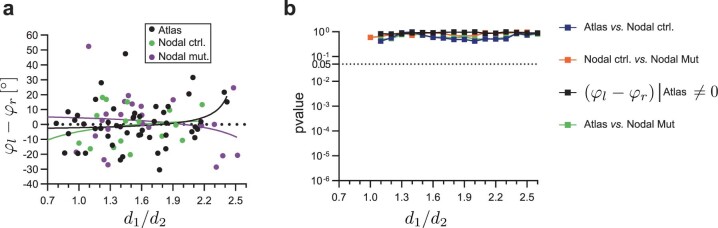
The inflow tract shows *φ* angle left-right symmetry. **a**, Plot showing the evolution of *φ* angles L-R symmetry depending on the *d1/d2* parameter. Curves represent Padé approximations for each experimental group. **b**, Evolution of p-values as a function of *d1/d2* for the deviation of the *θ* angle from L-R symmetry and for the comparisons between experimental groups, as indicated. A mixed linear model was applied to estimate the asymmetry between both sides at different stages (fixed effects) adjusted by the angle with two-sided p-value calculation. Source data

Heart tube looping is considered the earliest morphogenetic expression of left–right asymmetry in a mammalian developing organ. Here, we identified obvious signs of heart looping in stages 9 and 10; however, we wanted to explore systematically whether less obvious asymmetries might be present before the looping stage. Studying both the whole specimen collection and the collection of MSGs, we detected a possible recurrent difference in the angle of insertion of the IFTs. To study this aspect in more detail, we defined the direction of insertion of the IFTs via polar coordinates in a local system of orthogonal axes in which the *xy* plane is transversal to the embryo, and the *z* axis is aligned with the embryo AP axis (Fig. 5a, Extended Data Fig. 5 and Methods). The direction of insertion of the IFTs is then defined by two angles: the *θ* angle and the *φ* angle (Fig. 5a). When studying the evolution of these angles in correlation to the staging parameter *d1*/*d2*, we found that the *θ* angle progressively diverged between the left and right sides (Fig. 5b,c, ‘Atlas’), while the *φ* angle, on average, remained symmetric (Extended Data Fig. 6a,b). The *θ* angle significantly deviated from symmetry starting at *d1*/*d2* equal to 1.55, which corresponds to group 6 (Figs. 3f and 5c). The asymmetry of the *θ* angle further increased during the looping stages (*d1*/*d2* ≥ 2) until reaching a 20° difference (Fig. 5b,c, ‘Atlas’).

Related to the different angle of insertion of the IFTs, we frequently observed that the insertion of the right IFT into the ventricular region forms an acute angle, whereas the similar position on the left side is characterized by a much smoother transition, often even accompanied by a small bulge connecting the ventricle to the IFT (Fig. 5g). In hearts at the looping stage, these asymmetric connections seem to favor the typical rightward ventricular displacement during looping (see the group 9 representative in Fig. 5g), as the right-side acute angle may act as a hinge, favoring rotation toward the right side, while the bulge on the left side may oppose a similar rotation toward the left side.

We concluded that before heart looping is obvious, and starting at linear heart tube stages, left–right morphological asymmetry builds up in the IFT region, manifested in the different angles of insertion of the IFTs.

### *Nodal* controls early cardiac left–right asymmetry

To study whether early IFT asymmetry is under the control of the genetic pathway that controls left–right specification in the mammalian embryo, we studied this feature in *Nodal* mutants, which show randomized looping direction^19,20^. For this, we studied a collection of 3D images from *Nodal* mutants and their control siblings acquired in the laboratory of S.M.M. First, we determined that the staging systems described here were transferrable between labs, despite the different imaging methods and genetic backgrounds (Methods). We next calculated the *θ* and *φ* angles of the control embryos of this second collection and found progressive asymmetry similar to that observed in the original collection (Fig. 5b). Examining the independent evolution of the left and right *θ* angles in both control embryo collections showed that both tend to diminish with time, but the right one does it at a higher pace than the left one, which produces the progressive asymmetry (Fig. 5d). We then studied conditional *Nodal* mutants (*Hoxb1*^*Cre/+*^; *Nodal*^*flox/Nul*^) using similar procedures. *Nodal* mutants also showed IFT *θ* angle-specific progressive asymmetry; however, the direction of the asymmetry was reversed with respect to that in control groups (Fig. 5b,c). While in controls, the left–right *θ* angle evolves from zero toward positive values, in *Nodal* mutants, the values evolve from zero toward negative values. This is due to a tendency of *θ* angle to reduce at a higher pace in the left than in the right side in the mutants (Fig. 5e). In all cases, the differences between *Nodal* mutants and the controls start to build up from *d1*/*d2* values >1.5–1.55. Our results show that early IFT asymmetry is related to heart looping, both in normal development and in mutants, affecting left–right specification (Fig. 5f).

### Early left–right asymmetry affects pericardial tissues

**Extended Data Fig. 7:**
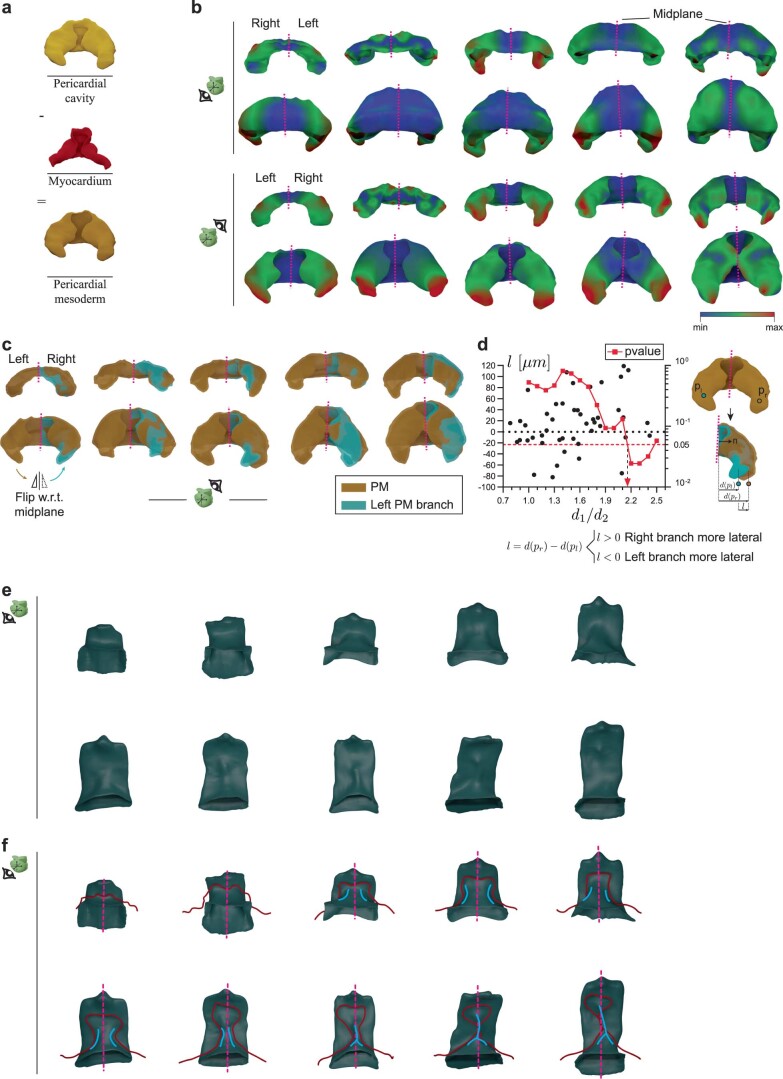
Signs of early asymmetry in the pericardial mesoderm. **a**, Reconstruction of the pericardial mesoderm. **b**, Maps of local deformation distribution resulting from mapping left and right sides of the pericardial mesoderm to each other. Specimens shown are the RSGs from ventral and dorsal views. Dotted magenta line indicates the midplane intersection. The color coding corresponds to the relative levels of morphological variation within each specimen. **c**, Ventral view of the pericardial mesoderm of RSGs (orange), including the view of the left side flipped with respect to the midplane onto the right side. **d**, Plot shows the characterization of the misalignment between the two sides of the pericardial mesoderm. A mixed linear model was applied to estimate the asymmetry between both sides at different stages (fixed effects) adjusted by the *l* distance with two-sided p-value calculation. The schemes on the right show how the *l* distance was determined. **e**, Ventral view of the foregut pocket endoderm in the RSGs. **f**, A similar representation of the foreguts in e, indicating the midplane intersection (pink dotted lines), the ridges detected, first bilateral and later fused medially (blue), and the *d1* line, at the border between the myocardium and the splanchnic mesoderm (red). **n**, normal direction of the midplane. Source data

To investigate whether and how the left–right asymmetries detected in the heart tube affect the pericardial tissues, we extended our study to the pericardial cavity mesoderm and the endoderm. To perform this study, we first determined the medial–sagittal plane of the embryo (Extended Data Fig. 4b) and determined the fitting between the left and right sides of the pericardial mesoderm (Extended Data Fig. 7a) by flipping one onto the other and quantifying the local deformation required for a perfect match of the geometries (Extended Data Fig. 7b). This study identified asymmetry of the lateral–posterior extensions of the pericardial cavity in which the IFTs are encased. A visualization of the fitting between the right side of the pericardial mesoderm and the flipped left side in the RSG collection suggested a systematic positioning of the left side closer to the midline plane in groups 6+ (Extended Data Fig. 7c). This was confirmed in a morphometric analysis of the whole specimen collection (Extended Data Fig. 7d). This left–right deviation coincides with the direction of the progressive asymmetry of the *θ* angle detected in the IFTs.

In the endoderm, we observed a deformation of the ventral foregut in correlation with the displacement of the splanchnic mesoderm layer during dorsal closure (Extended Data Fig. 7e). In early specimens, this deformation has the form of two bilateral ridges that coalesce into one, as the two splanchnic mesoderm sides reach the midline (Extended Data Fig. 7f). From RSG7+, this ridge shows a deviation from the midline such that its anterior side displaces rightward, and its posterior side displaces leftward. This displacement is concomitant with a similar tilting of the closing borders of the splanchnic mesoderm, which produces an asymmetric mesocardium, as previously described^11^. The observations are compatible with the deformation of the endoderm due to the movement of the splanchnic mesoderm over its ventral surface.

### A dynamic atlas of the embryonic heart

**Fig. 6:**
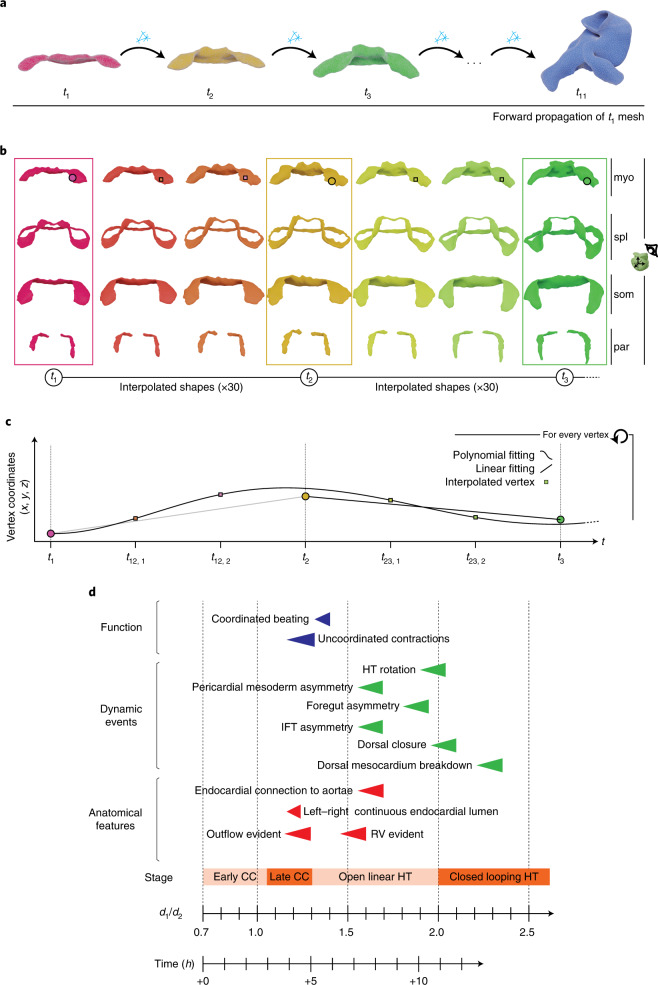
Dynamic meshes defining the full spatiotemporal domain of heart tube morphogenesis. **a**, Elaboration of a continuous sequence using the MSG shapes. The process starts by mapping the mesh connectivity from the first time point to the next shape. This process is repeated until the connectivity of the first mesh is propagated until the final mesh at *t*_10_. This process can also be applied using the RSG shapes. **b**, Concept of shape interpolation applied to the three first time points of the RSG shapes. Each row shows the result for the different tissues forming the pericardial cavity. The dots depicted on top of the myocardium shape illustrate the position of a single vertex traveling through the shapes as morphogenesis progresses. **c**, The plot illustrates the estimated trajectory of the vertex highlighted in **b**; myo, myocardium; spl, splanchnic mesoderm; som, somatic mesoderm; par, paraxial mesoderm. **d**, Temporal reference for morphological and physiological events during heart tube formation. Data on the onset of contraction/beating and real-time scale were obtained from in vitro live-imaging videos^50^; HT, heart tube; RV, right ventricle.

To simulate temporal evolution through the stages characterized, we used the 10 MSG shapes generated plus specimen 51 (Supplementary Fig. 5) and the method described in ref. ^18^ to generate a smooth temporal transformation between stages (Fig. 6a). To achieve this, we propagated a common mesh connectivity, identifying equivalent vertices from the first to the last shape (Fig. 6a). Once the same mesh connectivity is set on all the MSGs, they were rigidly aligned to the first mesh (*t*_1_). In addition, a second alignment step was taken using the landmarks to register equivalent landmarks across stages. We then applied an interpolation technique (Methods) to estimate the positions of each vertex between consecutive MSGs so that transitions between positions take place smoothly (Fig. 6c) and inserted 30 interpolated shapes between each MSG. This approach generated a dense temporal sequence of 3D meshes of heart tube morphogenesis (Supplementary Video 1). The model generated does not represent the evolution of a concrete specimen but a most probable trajectory of the morphology of the forming heart tube based on averaging the shapes of several hearts at each developmental time.

This approach did not allow us to include the models of the rest of tissues associated with the forming heart tube because mismatches are generated at the limits between the MSGs of the different tissues. To generate a dynamic model that includes all the tissues, we then used the collection of 10 RSGs (Fig. 6b and Supplementary Videos 2–7). Given that these are specific specimens, their matching to the surrounding tissues is improved. This model represents a possible evolution of the morphology of the heart tube and its associated tissues based on different specimens that represent consecutive stages of heart development. Examples of the interpolated tissues are provided in Fig. 6b.

Finally, we correlated previously described stages of heart tube development and several morphological and functional landmarks of cardiac development to the evolution of *d1*/*d2* (Fig. 6d) and incorporated this information to the videos of the four-dimensional models (Supplementary Videos 2–7).

## Discussion

Here, we generated a comprehensive dynamic and realistic representation of cardiac tissues during primitive heart tube formation in a mammalian embryo. In the process of generating a unified dynamic model using information from a collection of specimens, we faced the problem of morphological variability during early cardiogenesis, which represented limitations to describing ‘normal’ development. We thus developed strategies to describe ‘most likely’ geometries for each developmental stage. To achieve this, we first developed a morphometric staging method independent of subjective appreciation of specimens, thereby improving the current state of the art in the field^10,11,14^. Unsupervised clustering and assessment of parameters that measure the quality of classifications identified a simple relationship (*d1*/*d2*) as the best staging parameter. While we used this parameter to group specimens in relation to their developmental progression, and this was essential to map variability and generation of the 3D+t model, the great advantage of using *d1*/*d2* is that it allows the assignment of specimens along a continuum of heart tube morphogenesis. We think this staging method will be of utility in the precise definition of cardiac developmental timing when comparing 3D data between different laboratories. Furthermore, we identified simpler measurements, like the height-to-width ratio, as very highly correlated with *d1*/*d2* and accessible to all labs and 2D–3D imaging approaches.

Ten groups were established according to the variation of *d1*/*d2*, which allowed for the statistical analysis of morphology within groups. The use of a recently developed method^18^ for surface comparison allowed the definition of mean shapes and the application of statistical analyses to each vertex, thereby generating maps of local variability. We propose that this approach is of high value when describing mammalian organogenesis because it simultaneously provides information about the ‘average’ geometry of a developing organ and about the regions that are more likely to vary from specimen to specimen.

Failures in early steps of cardiac development can lead to misalignment of the chambers and great vessels or to different types to left–right cardiac mispatterning. In particular, we identified strong variability at the dorsal borders of the myocardium. These dorsal borders, initially placed laterally, end up fusing at the midline, forming the dorsal mesocardium, which later disappears during looping. Important mechanical roles have been proposed for the mesocardium during looping^11^, and the mesocardial myocardium later localizes to the inner curvature of the looping heart, which plays essential roles in the alignment of the cardiac chambers and the great arteries (reviewed in ref. ^21^). The finding of high variability in this region, therefore, could be related to the onset of malformations associated with the mesocardial/inner curvature morphogenesis. Other regions of high variability were the IFTs, which correlate with the highly dynamic and transient nature of these structures. Finally, apical regions of the future left ventricle also show high variability, possibly associated with asynchrony of the ballooning process^22^ between specimens.

Our approach also allowed us to study the left–right symmetry of the forming heart tube. Heart tube looping is considered as the earliest morphological evidence of organ left–right patterning in the embryo. In particular, cardiac left–right patterning defects are commonly associated with impairing congenital heart disease. Here, we describe an aspect of left–right asymmetry in the forming heart affecting IFTs and preceding heart looping. The influence of the IFT region in heart looping has been previously proposed, mainly through the exertion of asymmetric forces from the left and right sides of the venous pole, provoked by differences in cytoskeletal contraction, cell migration and/or proliferation^23–25^. We therefore identified the earliest signs of morphological asymmetry in the mouse embryo localized to the IFTs of the forming heart. This asymmetry precedes looping and is likely to affect this process, as any active or reactive force exerted by/on structures oriented in different angles is predicted to produce asymmetric forces. In agreement with this view, *Nodal* mutants, which show a high incidence of heart looping defects, show reversed asymmetry of the IFT insertion angles. Nonetheless, there is no strict relationship between IFT insertion angles and the looping direction; only 50% of *Nodal* mutants show reversed direction of looping^19,20^, while some of the wild-type specimens show negative left–right *θ* angles during normal looping. We therefore propose that left–right bias in the orientation of the IFTs depends on the general left–right patterning mechanisms of the embryo and imposes an architectural bias to the forces that determine heart looping direction in interaction with additional determinants. Interestingly, we also found left–right alteration of the pericardial cavity and endoderm, suggesting that the process of heart looping involves a wider asymmetry also affecting the peripheral tissues. Our observations on the relationship between splanchnic mesoderm movements and ventral foregut deformation strongly suggest that the splanchnic mesoderm has an active role in generating left–right asymmetries in the pericardial cavity. In this respect, the described transient expression of *Nodal* in the left splanchnic mesoderm and its influence on cell proliferation and matrix remodeling^20^ might represent an important factor in the generation of left–right asymmetry in the pericardial cavity.

The dynamic models generated here represent highly detailed atlases of the different tissues involved in primitive cardiac tube formation. These atlases will be useful for the combination of relevant biological information produced from different experiments or labs into a unique spatial representation. This opens the door for establishing a common space where scientists can share analyses on quantitative aspects representable in the spatiotemporal domain, for example, gene expression patterns. Furthermore, the atlas generated is embedded in a statistical framework that accounts for natural variability, which can be used as a reference for analysis of mutant embryos, either at global or local scales.

Deep understanding of morphogenesis involves identification of the sources and the temporal and spatial distribution of forces that shape developing tissues. Predictive modeling is ultimately required to fully acquire this knowledge^20,26^; however, it critically relies on access to realistic dynamic models of the geometry of the tissues/organs under study^6^. The dynamic model developed here will be useful for the development of finite-element studies that explain the forces that drive cardiac tube formation, one of the most complex morphogenetic processes in the mammalian embryo, which underlies the high incidence of cardiac malformation in newborns.

## Methods

### Animal model

The following are mouse alleles used in the study: *Mesp1*^*Cre*^ (ref. ^27^; MGI:2176467), *Rosa26R*^*mTmG*^ (ref. ^28^; MGI:3716464), *ROSA26R*^*Tomato*^ (ref. ^29^; MGI:6260212), C57BL/6 (Charles River), *Nodal*^*flox*^ (ref. ^30^; MGI:3056345)*, Nodal*^*nul*^ (ref. ^20^) and *Hoxb1*^*Cre*^ (ref. ^31^; MGI:2668513). Embryos imaged to generate the M.T. collection were on a mixed 129/SvJ × CD1 genetic background. Embryos imaged to generate the S.M.M. collection were on a mixed C57BL/6 × 129/SvJ genetic background. Adult mice were housed in an air-conditioned room with a 12-h light/12-h dark cycle and free access to water and chow diet. A total of 4 males and 10 females were used as progenitors for embryo collection. Animal procedures in the M.T. laboratory were approved by the CNIC Animal Experimentation Ethics Committee, by the Community of Madrid (reference PROEX 144.1/21), and conformed to EU Directive 2010/63EU and Recommendation 2007/526/EC regarding the protection of animals used for experimental and other scientific purposes, enforced in Spanish law under Real Decreto 1201/2005. *Nodal* mutants were housed in the Laboratory of Animal Experimentation and Transgenesis of the SFR Necker, Imagine Campus, Paris. Animal procedures were approved by the ethical committees of the Université de Paris and by the French Ministry of Research.

### Embryo preparation

Embryos were collected from 00:00 to 08:00 (E8–E8.33). Pregnant mice were killed by CO_2_. Embryos were dissected, and the amnion, atlantois and yolk sac were kept, fixed overnight in 2% paraformaldehyde at 4 °C, washed three times in PBS and permeabilized in PBS 0.5% Triton X-100 for 30 min. Embryos were incubated overnight at 4 °C in PBS with DAPI (1:500) and washed in PBS before embedding in low melting point agarose. Once the agarose polymerized, using a scalpel, a small agarose cube was created (0.5 cm × 0.5 cm × 0.5 cm) containing the embryo. The cube was then immersed in CUBIC I (ref. ^32^) at 4 °C over 3 days, with CUBIC I renovation after the first day. Whole-mount *Nodal* mutants and their controls were fixed at E8.5 in 4% paraformaldehyde and dehydrated in 100% methanol. Embryos were then stained with Hoechst as a nuclear counterstain and transferred to R2 CUBIC clearing reagents.

### Embryo imaging

Embedded embryos in the laboratory of M.T. were imaged on an inverted microscope through an 80-µm-thick cover glass placed on a perforated culture plate to minimize optical pathway length (Fig. 1a). Given the high sensitivity of CUBIC I to water concentration, a water-saturated atmosphere was created to prevent evaporation. Confocal images were obtained using an SP8 Leica confocal microscope and an HC PL Apo CS2 20 0.75-NA multi-immersion lens corrected to glycerol immersion medium. The following scanning settings were used: *xyz* mode, bidirectional, 400 Hz; pinhole, 56.7 µm; zoom, 1.15; laser lines used, diode 405 nm (5% power, DAPI signal), 488 nm (power 15%, mGFP signal) and 561 nm (20%, Tomato signal); detectors used, PMT (424 nm–474 nm), HyD (496 nm–529 nm, gain 100% and 583 nm–667 nm, gain 170%). For large specimens, a tile scan was performed, and tiles were stitched using Leica LAS X 3.5.2.18963 software. For the *Nodal*-mutant and control series, 16-bit images were acquired with a Zeiss Z.1 light-sheet microscope and a ×20/1.0 objective.

### Image visualization

Visualization of image stacks was performed in ImageJ 1.53f (ref. ^33^) and ITK-SNAP 3.8.0.

### Image processing

Raw images were deconvolved with ‘Huygens Professional version 19.10’ to compensate for the blurring and noise inherent to the optical system. Deconvolved images were exported in tagged image file format, rectified for axial orientation and saved in NIFTI format. All the image processing steps described hereafter were implemented in Python 3.8 (ref. ^34^) making use of different libraries for each specific process. The library NiBabel, version 3.1.1 (ref. ^35^), was used for reading and writing NIFTI images. Images read in this format are represented as array-like structures, defined as an instance of the class ndarray, which is part of the scientific computing package NumPy^36^. The library used for processing the segmentation images (Fig. 1f) was scikit-image, version 0.16.2 (ref. ^37^).

### Image segmentation

Segmentations were performed using ImageJ in a scaled-down version of the images by applying a diminishing scale factor equal to 2 in each of the three axes. This resulted in lighter files, with *xy* pixel sizes of 0.76 µm–0.98 µm and a *z* step of 0.98 µm–4 µm, depending on the specimen. The reduced resolution did not introduce loss of accuracy in the segmentation at the tissue level, because the segmented tissue layers are approximately 20 µm thick. For segmentation of the tissues forming the pericardial cavity and the endocardial lumen, we used the open-source multiplatform ITK-SNAP^38^. Automatic segmentation based on intensity levels was applied to the Tomato signal, which was present ubiquitously within mesodermal cells. First, we activated the active contour tool and defined the region of interest as the cardiogenic region. Then, we used thresholding as presegmentation mode and adapted the threshold mode and lower and upper values. This creates a new synthetic image called the speed image, which has values close to 1 inside the structure of interest. In the next step, we placed several seeds within the domains of the structure of interest, distributed across the cardiogenic mesoderm. Then, the segmentation was initialized and the seeds extended across the structure of interest. We then performed expert manual delimitation of each of the tissues composing the pericardial cavity and the myocardium and correction of failures detected in the automatic segmentation. For this task, both the mGFP signal, which labels all mesodermal cell membranes, and the Tomato signal, which labels all cell bodies, were used.

The segmentation of the forming aortae and the foregut pocket lumen was done using Mask R-CNN^39^. The network was trained for the 2D detection of both, the forming aortic lumen and the closed contour defined by the foregut endoderm (as is shown in Fig. 1d). The elaborated dataset was composed of 351 slices taken from random specimens and random planes. In each slice, the contour of every different instance was annotated using ImageJ’s ROI manager. The dataset was divided 80/20 for training and validation, respectively. The training and detection were conceived for detection in slices from the frontal view along the ventral–dorsal axis. The performance achieved for the detection of the aortae and foregut pocket was 88% and 91%, respectively. The foregut endoderm was derived from the segmentation of the foregut pocket lumen, performed by selecting negative levels for every channel (Fig. 1d). First, the segmentation image was dilated using the library Scipy, version 1.5.3, using a multidimensional binary dilation. This resulted in an expanded segmented image that covers the surrounding foregut endoderm. Finally, the original segmentation of the foregut pocket was subtracted from the dilated segmentation, resulting in a new segmentation image only representing the foregut endoderm. To determine the limit of the foregut endoderm extension in a reproducible manner across all the specimens, we defined a sphere, centered at the anterior tip of the foregut pocket, with a radius equal to the distance to landmark lm12 (Extended Data Fig. 4c) and calculated the intersection with the foregut endoderm mesh.

### Three-dimensional reconstruction

After the 2D segmentations were complete, we used the marching cubes algorithm with the library PyMCubes, version 0.1.2, to obtain 3D reconstructions. The resulting meshes were then saved as polygon file format. The meshes were handled in Python by making use of the library Trimesh, version 3.9.19, which was used to filter the meshes, thus creating smooth surfaces. As part of the postprocessing steps, meshes for each of the tissues segmented were first simplified using a simplify quadratic decimation algorithm provided by the library Open3D, version 0.7.0 (ref. ^40^), to a number of faces equal to 10,000. Then, meshes were checked to make sure that they are closed surface of genus 0 by using Trimesh 3.9.19 and visual inspection using Meshlab 2020.12. The reconstructed surfaces were flipped with respect to the *y* axis of the image (Fig. 1b), the reason being that acquisition in the confocal microscope generates left–right inverted images. By doing this, all surface meshes are oriented as indicated in the system of reference shown in Fig. 1a.

### Embryo collection

We imaged a total of 52 specimens. All specimens were temporally ordered according to *d1*/*d2* except two specimens (E51 and E52) that were manually assigned because they represented outliers in the classification (Supplementary Figs. 1–8) and one specimen (E3) in which the partial differentiation of the CC did not allow for the calculation of *d1*/*d2* and was manually assigned to group 1 by similarity.

Within the collection, there are some exceptions in which we were not able to obtain all the tissues and shapes of interest (Fig. 1a). We list here the name of the embryo, its corresponding staging group and what makes the dataset exceptional.

- E21, group 5: unable to reconstruct the somatic or paraxial mesoderm of this embryo.
- E22, group 5: unable to image the most dorsal part of the foregut endoderm and the aortic lumen in that region.
- E26, group 6: unable to fully reconstruct the foregut endoderm.
- E37, group 8: only myocardium and the endocardial lumen could be reconstructed.
- E41, group 8: unable to segment the splanchnic and somatic mesoderm.
- E51, outlier: unable to segment the foregut endoderm and the aorta.

### Midsurface extraction

The meshes were first isotropically remeshed^41^ to a 2-µm edge length and smoothed^42^. Then, the skeleton of the mesh was derived using ref. ^15^ and smoothed to eliminate spikes and/or high-frequency details. The former step was repeated until the algorithm created a monolayer mesh. The resulting mesh was then resampled using a Poisson disc sampling and reconstructed using the Ball pivoting algorithm^41^. After this, the mesh was smoothed and postprocessed to eliminate imperfections (holes, non-manifold edges, non-manifold vertices and so on). The final state of the midsurface comes given by a last isotropic explicit remeshing (2 µm).

### Calculation of distances on the surface of the tissues

In general, distances between a pair of landmarks were calculated as the geodesic distance between the vertices representing each landmark. The library used for these computations was Networkx, version 2.4 (https://networkx.org). In the cases where the distance between landmarks was defined along the edge of the shape (for example, *d1* and *d2*; Fig. 3a), the distance was calculated by first projecting the landmarks on the midsurface. Then, for each of these points, its closest vertex to the outline (Fig. 3a) was determined. Finally, the geodesic distance was calculated as the accumulated sum of the length of every edge defining the outline between both points.

GraphPad Prism 8.0.1 was used for data representation and statistical analysis.

### Calculation of the dorsal closure gap

Landmarks 9 and 7 and 8 and 10 were used to define the edges of the dorsal lips of the myocardium (labeled as yellow in Extended Data Fig. 4a). The dorsal gap, *l*, measures the length of the shortest line that joins both lips. The calculation of *l* was achieved by the subdivision of the edge of the lips, generating *N* points in the left side and *K* points in the right side. Then, for every point *j* (left lip) to any point on the right lip, the minimum distance was calculated, *d*(*p*_*j*_, *q*). Finally, the minimum of the calculated distances was taken as the dorsal gap.

### Surface map computation

We computed surface maps using the method described in ref. ^18^. This method takes two topologically equivalent surface meshes and a sparse set of corresponding points (landmarks) as input. As output, it produces a continuous and bijective (1:1) map of correspondence between the vertices of the two surfaces. The algorithm minimizes intrinsic mapping distortion, which promotes the alignment of geometrically similar surface regions^43^. Maps are initialized through the layout embedding method presented in ref. ^44^. To establish correspondence between different tissue meshes, we established a sparse set of corresponding points (landmarks) as inputs (Fig. 4a(i) and Extended Data Fig. 4c–f). We provide a software package that implements these procedures, including the vertex-to-vertex mapping tool (SurfaceMapComputation) and a visualization tool (ViewMap) that displays a rigid registration of the shapes. This allows for the adjustment of the landmarks used, the addition of new landmarks and/or trying different initialization algorithms for finding the best mapping between two shapes. The software takes as input parameters the two shapes to be compared, a minimum of four landmarks, number of iterations and a choice of algorithms for the initialization of the map. The initialization algorithm can be automatically selected by the software or manually selected from those described in refs. ^44,45^.

### Shape averaging

We selected one reference shape per group (Fig. 4a(i)). We then computed surface maps^18^ from the reference to all other shapes of the group. We used each map to transfer the vertices of the reference mesh to the respective target surface, establishing a vertex-to-point correspondence. Using this correspondence, we rigidly aligned all shapes of the group to the reference shape to compensate rotational and translational misalignments. We then computed the average 3D position per vertex of the reference mesh, that is, the mean of the reference vertex itself and its corresponding points on the other rigidly aligned shapes (Fig. 4a(ii)). As a measure of local shape variability within the group, we report the standard deviation per set of corresponding vertices given by the square root of the largest eigen value of the covariance matrix (Fig. 4a(ii)).

### Identification of medoid specimens

**Extended Data Fig. 8:**
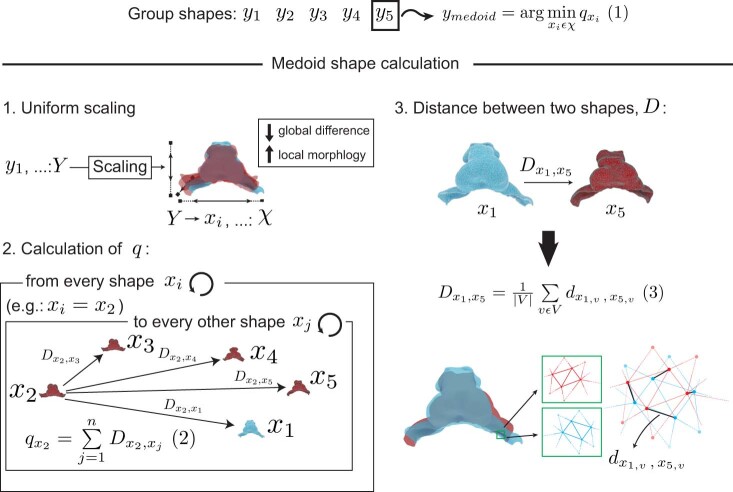
Details of the calculation of the medoid shape of a group. Figure shows the steps to determine the medoid shape of a group, following the terms included in equation 1. Shapes named and enumerated in the figure correspond to group 8. ***y***, indicates an individual shape of the group; ***Y***, indicates the set of shapes in the group; **x**_**i**_, indicates a shape of the group, after uniform scaling; ***X***, indicates the set of shapes in the group after uniform scaling; **q**, indicates the accumulated sum of morphological distances between a shape and all the other in the group; ***n***, number of shapes in the group; ***D***, morphological distance between a pair of shapes; ***v***, index of a specific vertex; ***V***, set of vertices.

The medoid shape of a group is represented by the geometry whose accumulated differences with respect to all others in the group is the smallest (Extended Data Fig. 8, equation 1). First, all the shapes in the group were uniformly scaled to minimize the difference in global size so as to prioritize local morphological variation versus size differences. Then, the accumulated difference *q* for every shape was calculated. This was done by adding all the morphological distances, *D*, between each shape and all the others in the group (Extended Data Fig. 8, equation 2). The morphological distance, *D*, between a pair of shapes was calculated as the average of all the distances between the corresponding vertices of the shapes, *d* (Extended Data Fig. 8, equation 3).

### Sequential mapping to generate the 3D+t atlas

We established vertex correspondence across the temporal sequence of shapes by computing a surface map between each consecutive pair of shapes. We started from the mesh of the earliest shape and successively connected the vertices to all meshes of the sequence (Fig. 6a). We then rigidly aligned all shapes of the sequence to avoid rotational and translational misalignments between time points. To achieve a high-quality surface approximation between time points, we used a remeshing technique based on ref. ^46^ to obtain a common triangulation that is suited for all keyframe shapes. We computed a target edge length field on each shape of the sequence^47^. We then used sequential surface maps to combine all fields via a vertex-wise minimum. In this way, if a shape in the sequence requires a high mesh resolution in some area, this demand will be translated to the whole collection in the common triangulation. We then iteratively improved the common triangulation by a series of edge splits, collapses, flips and tangential Laplacian smoothing^46^ and simultaneously considered the reference mesh embedded on all shapes of the sequence^48^. Finally, to approximate a continuous morphological evolution, we uniformly interpolated 30 new time points between each pair of consecutive shapes in the sequence. We initially determined the intermediate shapes via linear interpolation and then applied a polynomial smoothing filter to elude sharp transitions^49^. We provide a collection of 11 time points plus 30 interpolated time points between keyframes, which represents a total of 311 temporal shapes.

### Surface map computation between shapes of different topology

The dorsally closed heart and the splanchnic mesoderm represent cases of torus topology (genus 1), while all others have sphere topology (genus 0). This represents a compatibility problem when computing surface maps across the entire sequence. Therefore, we developed a methodology to turn these shapes into sphere topology surfaces by cutting the closed surfaces in a specific region. In the case of the dorsally closed heart, the border between the two myocardial lips is considered as the cut trajectory (Fig. 4b, yellow dotted line and arrow). In the case of the splanchnic, a cut is made along the anterior–posterior axis in the medial domain anterior to the OFT (Fig. 1g, black dotted line and arrow). For the latter, this procedure was done to all the splanchnic shapes of the collection because they all present genus 1 topology.

We processed each cut by individually triangulating the two emerging holes, yielding a closed surface of genus 0. To fuse any visible gap, we slightly extruded both newly triangulated areas (producing a subtle self-intersection of the geometry) and finally remeshed the affected regions.

### Definition of the IFT insertion angles

The insertion angles are the result of expressing the direction of the IFTs (*v*) via polar coordinates in the system of reference (Fig. 5a). The *θ* angle (Fig. 5a, middle) is defined within the *xy* plane, which normal direction is approximately equal to that of the dorsal–ventral axis of the embryo. To obtain comparable values of *θ* from both IFTs, we flipped the *x* axis of the reference system when computing angles for the right IFT. The *φ* angle (Fig. 5a, right) is defined as the angle through which *v* swings with respect to the cranio–caudal axis. This angle was calculated as the angle formed between *v* and the *z* axis of the reference system. As this angle is defined, it would oscillate around a value of 90°, so we applied an offset of −90° indicated by $0_{ + 90}^o$.

### Application of the staging method between different laboratories

**Extended Data Fig. 9:**
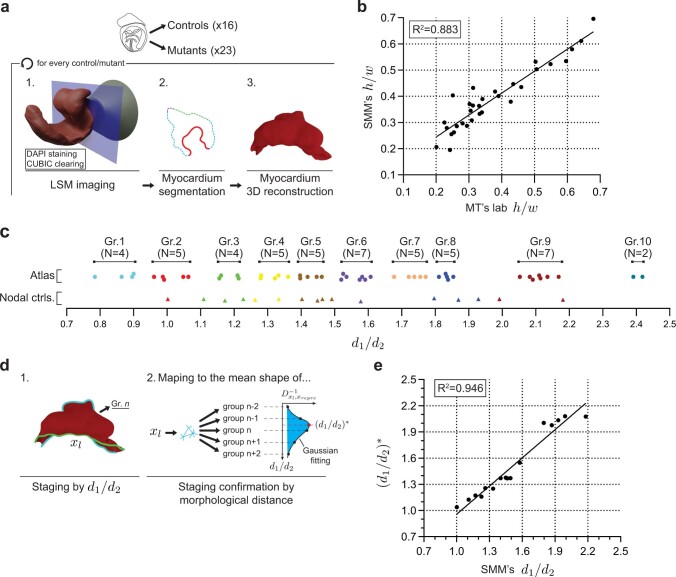
Pipeline for staging the specimens from the study of *Nodal* loss of function. **a**, Steps taken for the image acquisition and segmentation of the *Nodal* mutant and control specimen series. **b**, Correlation between the *h/w* staging parameter independently measured in the SMM and MT labs. **c**, Mapping of the *d1/d2* values measured in SMM’s control specimen collection. **d**, Procedure for the morphological mapping of the SMM’s control specimen collection to the atlas MSG collection. **e**, Correlation between the experimentally determined *d1/d2* parameter and the predicted *d1/d2* obtained from the morphological mapping of the SMM’s control specimen collection to the atlas MSG collection. Source data

The S.M.M. collection of control embryos was independently staged using the *h*/*w* parameter in the S.M.M. and M.T. labs. While the S.M.M. lab used Imaris 9.5.1 on the raw images, the M.T. lab segmented the images and measured on the segmentations. The correlation obtained between the two measurements was very high, indicating that different observers obtained similar measurements from differently treated images (Extended Data Fig. 9b). Next, the M.T. lab calculated *d1*/*d2* for each of the specimens (Extended Data Fig. 9c). Independently, the specimen segmentations were reconstructed and compared to each of the MSG collection via surface map computation (as in Extended Data Fig. 8b), which provided a position for each specimen along the MSG sequence with an estimated *d1*/*d2* value (Extended Data Fig. 9d). For *d1*/*d2* estimation, the morphometric distances were calculated between the specimen and the MSG of the group with a closest *d1*/*d2* value plus the two earlier and later MSGs in the temporal sequence (Extended Data Fig. 9e, part 2). The representation of the inverse of these distances (dependent variable) by the *d1*/*d2* values of the mean shapes (independent variable) was fitted to a Gaussian curve (Extended Data Fig. 9e, part 2). The *d1*/*d2* value corresponding to the peak fitted curve was identified as the predicted value for the specimen. In this way, we compared for each specimen of the control S.M.M. collection the directly measured *d1*/*d2* parameter to the *d1*/*d2* parameter estimated from the matching of the specimen morphology along the MSG sequence. This comparison resulted in a very high linear correlation between the measured and the predicted *d1*/*d2* values (Extended Data Fig. 9e). This shows that the *d1*/*d2* parameter and its close surrogate *h*/*w* can be used to stage collections of embryos of different genetic backgrounds and can be imaged/measured by different methods and laboratories.

### Renderings

All the renderings (figures and movies) have been processed in Blender 2.92.

### Reporting Summary

Further information on research design is available in the Nature Research Reporting Summary linked to this article.

### Supplementary Information

Supplementary Information Supplementary Figs. 1–8.

Reporting Summary

Supplementary Video 1 Continuous model of the myocardium, elaborated with the myocardial MSGs.

Supplementary Video 2 Continuous model of the myocardium, elaborated with the myocardial geometry of RSGs.

Supplementary Video 3 Continuous model of the splanchnic mesoderm, elaborated with the splanchnic mesoderm geometry of RSGs.

Supplementary Video 4 Continuous model of the somatic mesoderm, elaborated with the somatic mesoderm geometry of RSGs.

Supplementary Video 5 Continuous model of the paraxial mesoderm, elaborated with the paraxial mesoderm geometry of RSGs.

Supplementary Video 6 Continuous model of the myocardium (red) in combination with the splanchnic mesoderm (blue), elaborated with the geometries of RSGs.

Supplementary Video 7 Continuous model of all the tissues forming the anterior celomic cavity, elaborated with the geometries of RSGs; myocardium, red; splanchnic mesoderm, blue; somatic mesoderm, green; paraxial mesoderm, purple.

Supplementary Video 8 Continuous model of the myocardium, elaborated with the myocardial MSGs. Lateral views.

Supplementary Video 9 Continuous model of the myocardium, elaborated with the myocardial geometry of RSGs. Lateral views.

Supplementary Video 10 Continuous model of the splanchnic mesoderm, elaborated with the splanchnic mesoderm geometry of RSGs. Lateral views.

Supplementary Video 11 Continuous model of the somatic mesoderm, elaborated with the somatic mesoderm geometry of RSGs. Lateral views.

Supplementary Video 12 Continuous model of the paraxial mesoderm, elaborated with the paraxial mesoderm geometry of RSGs. Lateral views.

Supplementary Video 13 Continuous model of the myocardium (red) in combination with the splanchnic mesoderm (blue), elaborated with the geometries of RSGs. Lateral views.

Supplementary Video 14 Continuous model of all the tissues forming the anterior celomic cavity, elaborated with the geometries of RSGs; myocardium, red; splanchnic mesoderm, blue; somatic mesoderm, green; paraxial mesoderm, purple. Lateral views.

### Source data

Source Data Fig. 3 Statistical source data for Fig. 3.

Source Data Fig. 5 Statistical source data for Fig. 5.

Source Data Extended Data Fig. 2 Statistical source data for Extended Data Fig. 2.

Source Data Extended Data Fig. 3 Statistical source data for Extended Data Fig. 3.

Source Data Extended Data Fig. 6 Statistical source data for Extended Data Fig. 6.

Source Data Extended Data Fig. 7 Statistical source data for Extended Data Fig. 7.

Source Data Extended Data Fig. 9 Statistical source data for Extended Data Fig. 9.

## Data Availability

Datasets with the original segmentations, the processed 3D models and the 3D+t models are available at ‘Mendeley Data’ at https://data.mendeley.com/datasets/t828xhg66k/1. We recommend opening with the open-source software ‘Paraview’ (www.paraview.org). Instructions for visualizing the 3D images and videos in ParaView with custom selection of tissues, coloring and viewpoint are provided with these datasets. Datasets with the source confocal images and original segmentations are available at the IDR repository at 10.17867/10000174.

## Code availability

The software package including SurfaceMapComputation and ViewMap is available at Zenodo at 10.5281/zenodo.6390818.
