# Supplementary material for: Pseudodynamic analysis of heart tube formation in the mouse reveals strong regional variability and early left–right asymmetry
Source: Nat Cardiovasc Res. 2022 May 16;1(5):504–17. doi: 10.1038/s44161-022-00065-1 (PMC11357989; doi:10.1038/s44161-022-00065-1)
Supplement: Supplementary file 1 — Supplementary Figs. 1–8. [file 44161_2022_65_MOESM1_ESM.pdf]

nature portfolio

https://doi.org/10.1038/s44161-022-00065-1

Supplementary information

Pseudodynamic analysis of heart tube formation in the mouse reveals strong regional variability and early left–right asymmetry

In the format provided by the
authors and unedited

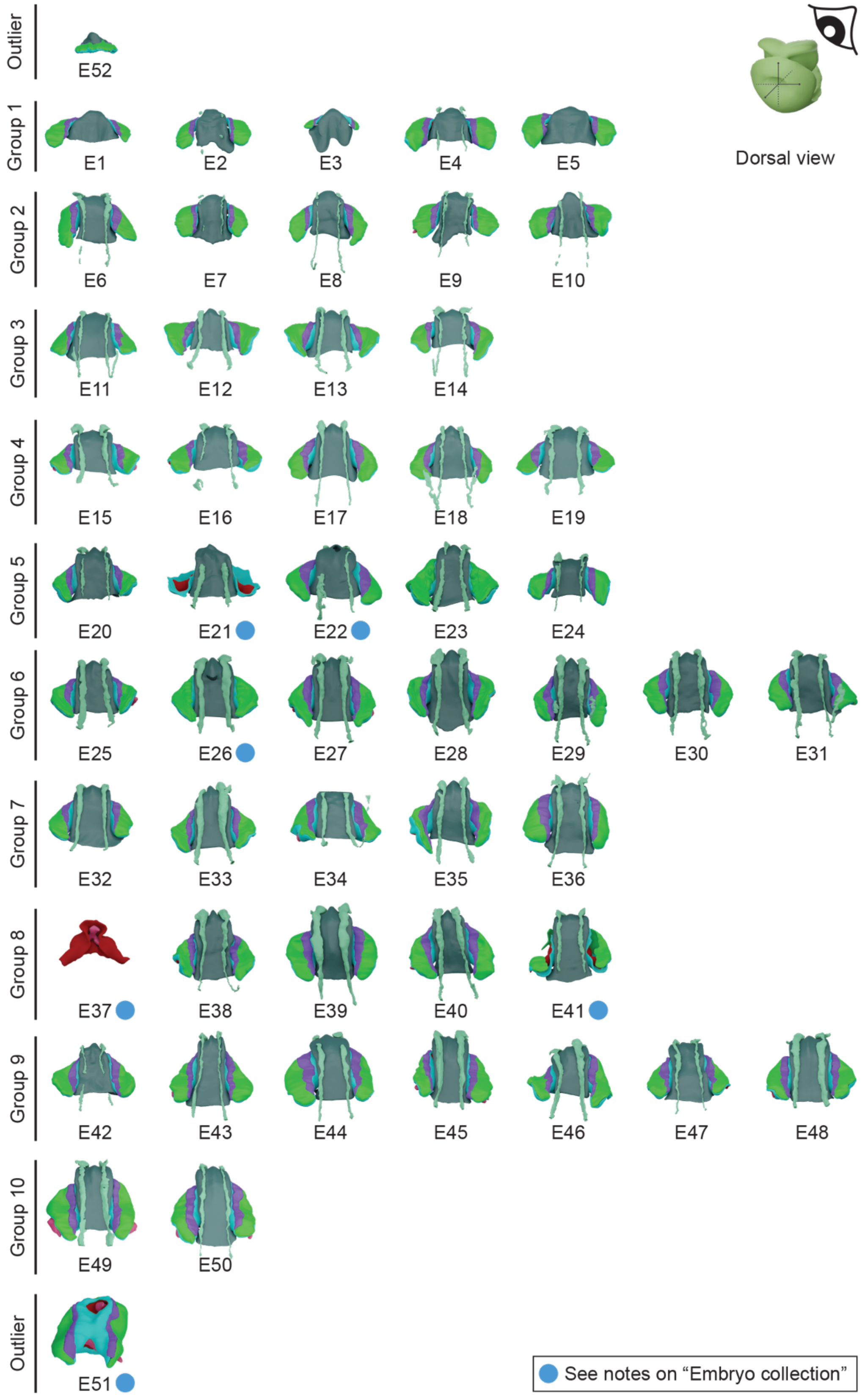

Supplementary Figure 1. Embryo collection classified by staging groups, showing a dorsal view of all tissues.

Dorsal view of all the specimens in the collection representing the processed surfaces of the complete set of tissues and shapes as described in Fig. 1g. Specimens E51 and E52 are outliers, left out of the stage classification.

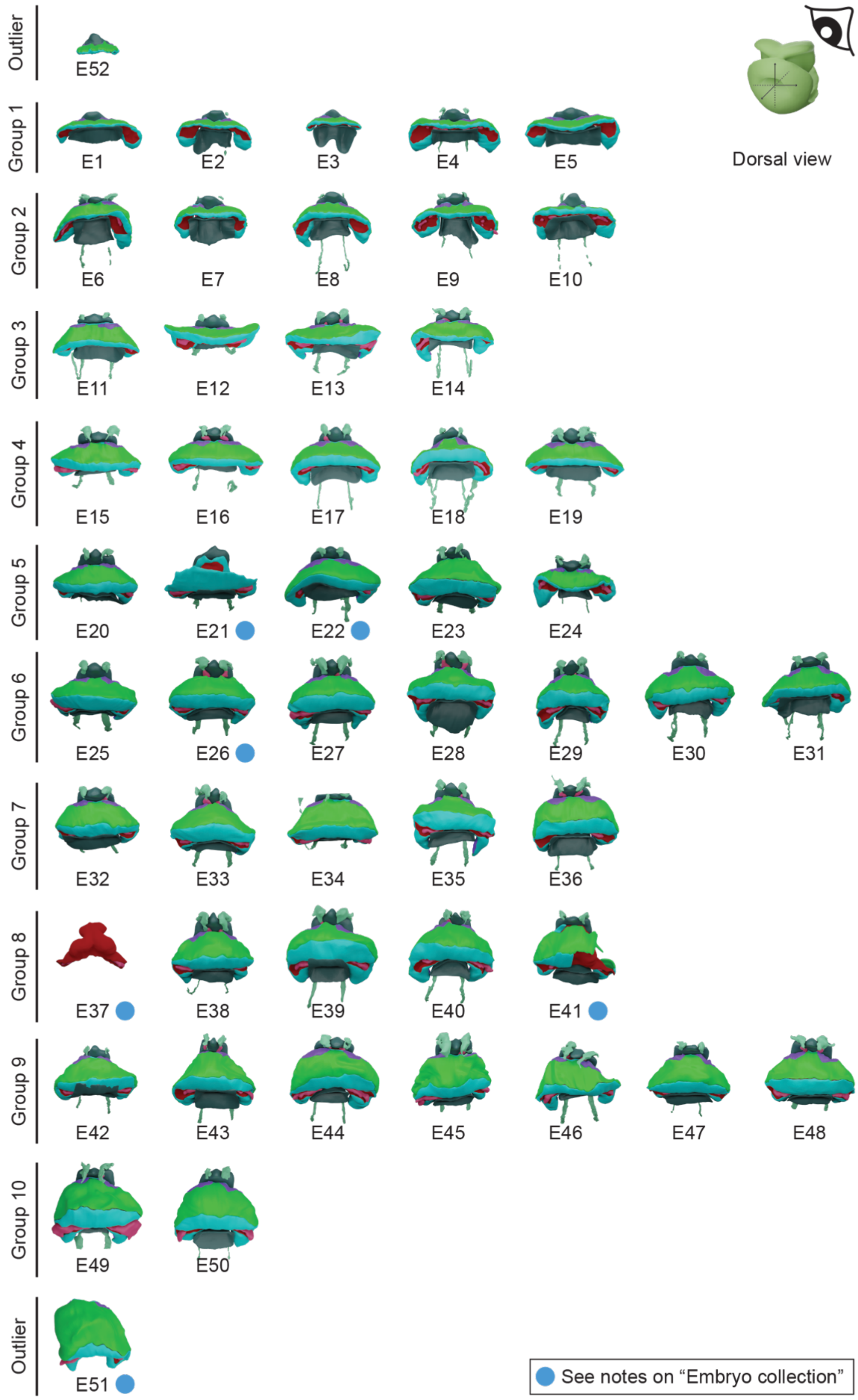

Supplementary Figure 2. Embryo collection classified by staging groups, showing a ventral view of all tissues.

Ventral view of all the specimens in the collection representing the processed surfaces of the complete set of tissues and shapes as described in Fig. 1g. Specimens E51 and E52 are outliers, left out of the stage classification.

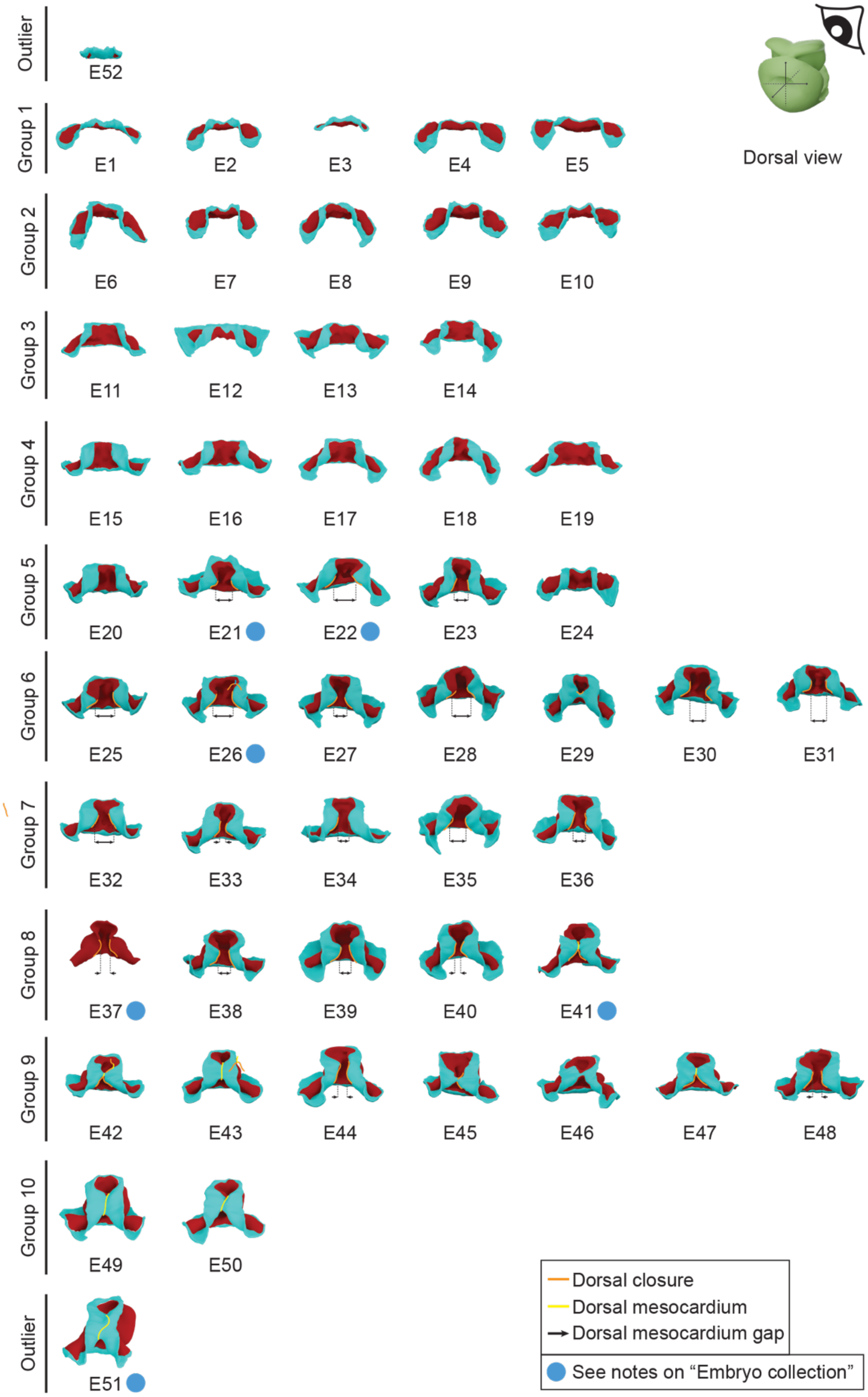

Supplementary Figure 3. Embryo collection classified by staging groups, showing a dorsal view of splanchnic mesoderm and myocardium.

Dorsal view of all the specimens in the collection representing the processed surfaces of the splanchnic mesoderm and myocardium. Specimens E51 and E52 are outliers, left out of the stage classification.

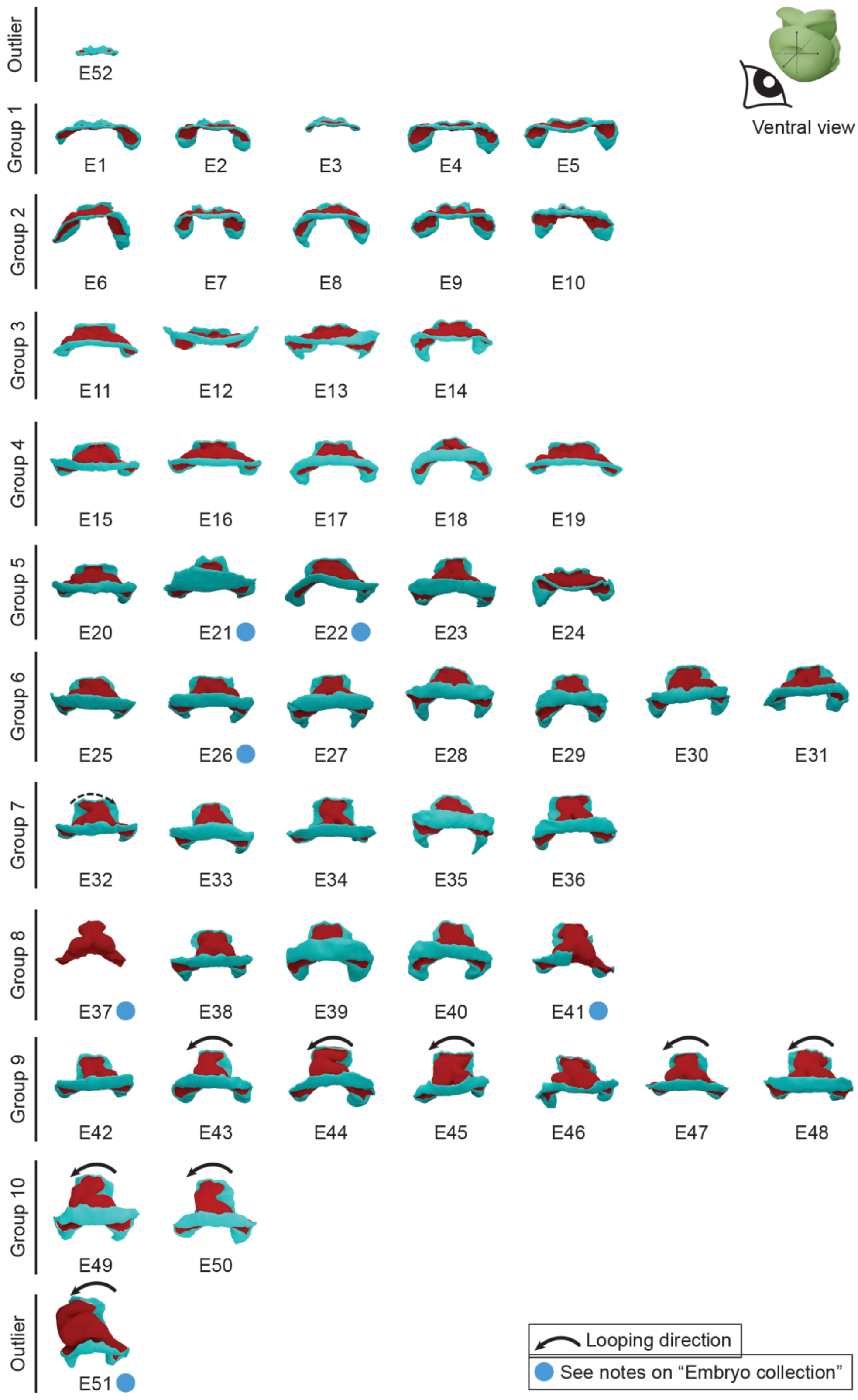

Supplementary Figure 4. Embryo collection classified by staging groups, showing a ventral view of splanchnic mesoderm and myocardium.

Ventral view of all the specimens in the collection representing the processed surfaces of the splanchnic mesoderm and myocardium. Specimens E51 and E52 are outliers, left out of the stage classification.

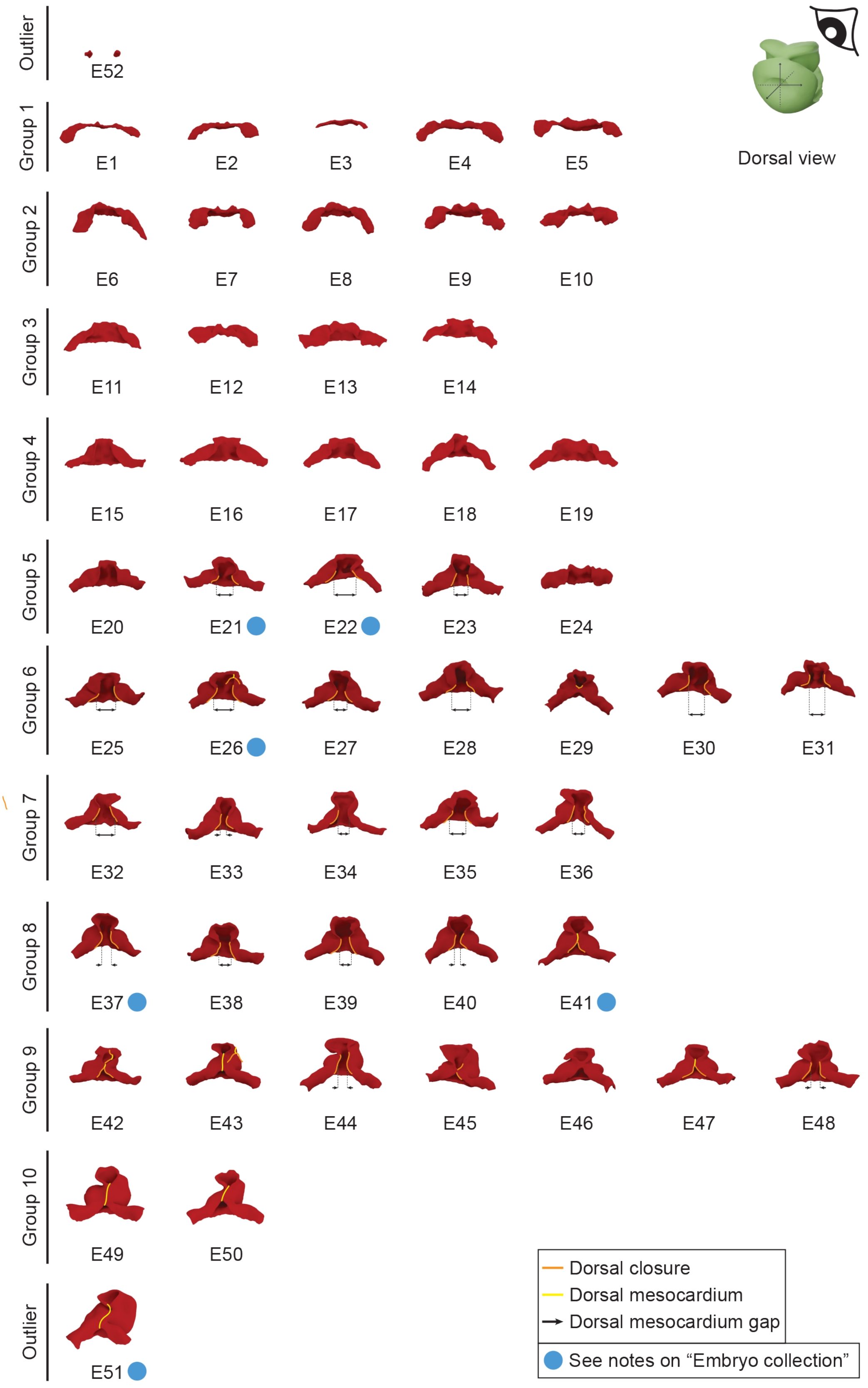

Supplementary Figure 5. Embryo collection classified by staging groups, showing a dorsal view of the myocardium.

Dorsal view of all the specimens in the collection representing the processed surfaces of the differentiated myocardium. Specimens E51 and E52 are outliers, left out of the stage classification.

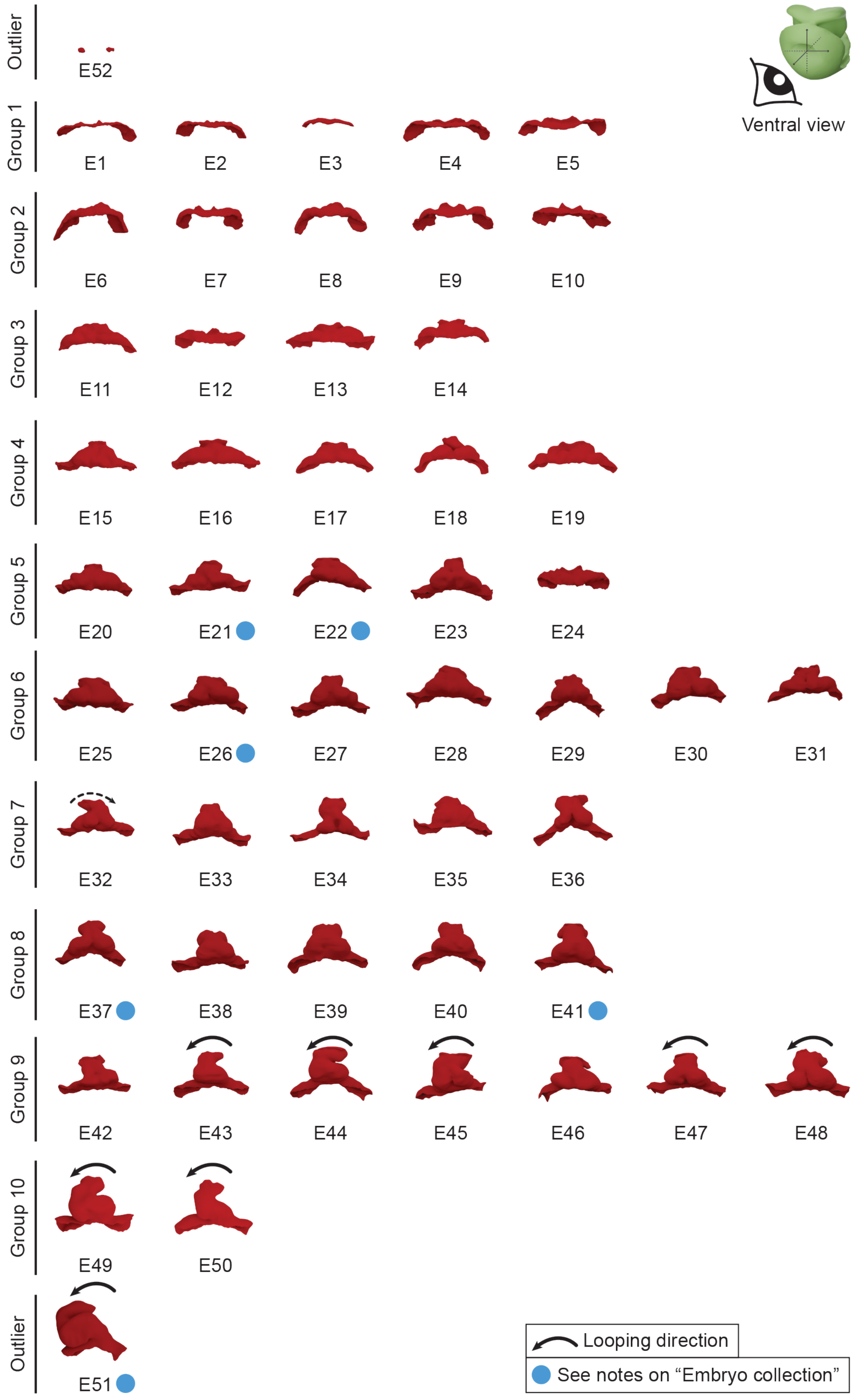

Supplementary Figure 6. Embryo collection classified by staging groups, showing a ventral view of the myocardium.
Ventral view of all the specimens in the collection representing the processed surfaces of the differentiated myocardium. Specimens E51 and E52 are outliers, left out of the stage classification.

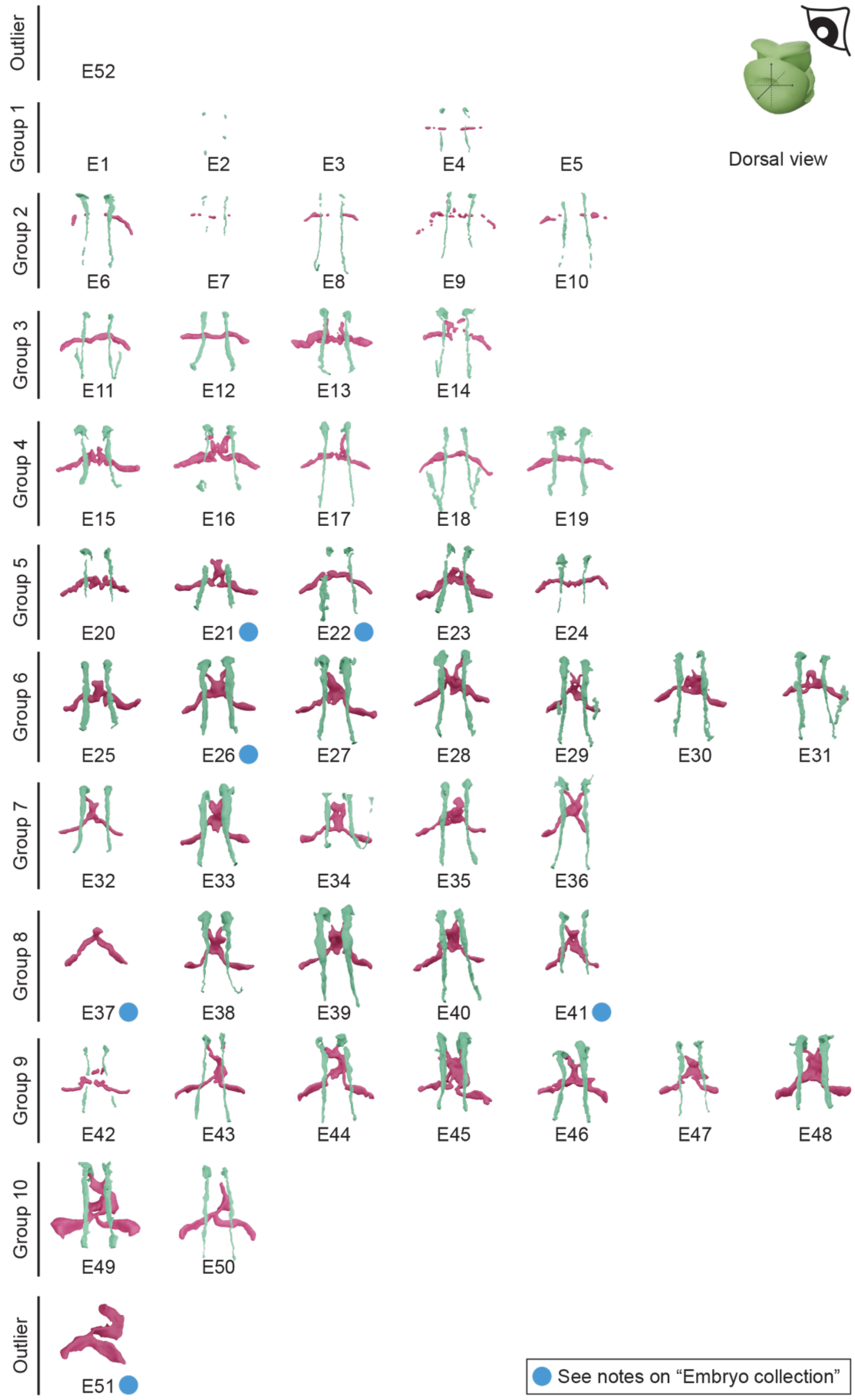

Supplementary Figure 7. Embryo collection classified by staging groups, showing a dorsal view of the circulatory system.

Dorsal view of all the specimens in the collection representing the processed surfaces of the circulatory system, split in two parts: endocardial lumen and aortic lumen. Specimens E51 and E52 are outliers, left out of the stage classification.

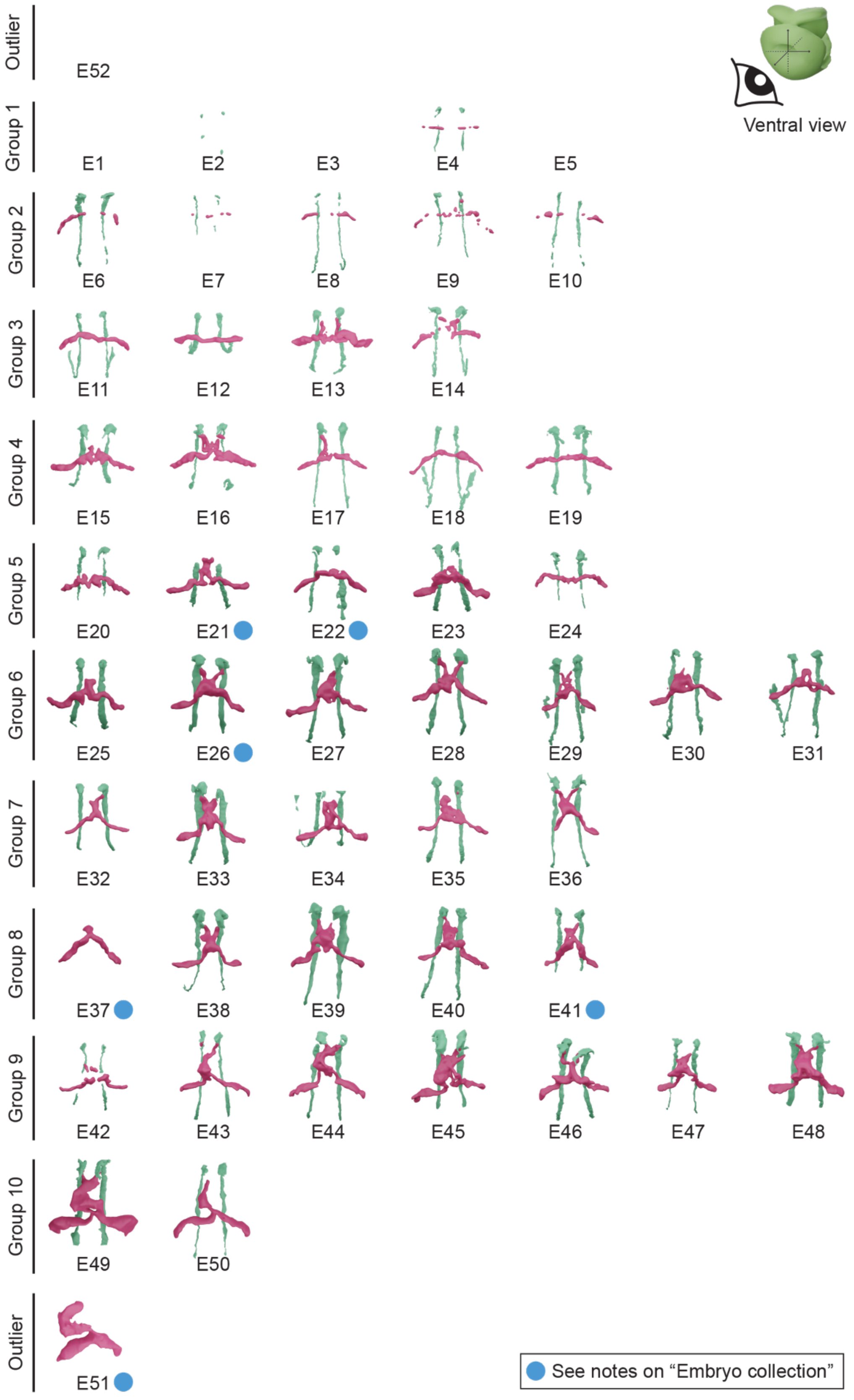

Supplementary Figure 8. Embryo collection classified by staging groups, showing a ventral view of the circulatory system.

Ventral view of all the specimens in the collection representing the processed surfaces of the circulatory system, split in two parts: endocardial lumen and aortic lumen. Specimens E51 and E52 are outliers, left out of the stage classification.
