# Supplementary material for: Pseudodynamic analysis of heart tube formation in the mouse reveals strong regional variability and early left–right asymmetry
Source: Nat Cardiovasc Res. 2022 May 16;1(5):504–17. doi: 10.1038/s44161-022-00065-1 (PMC11357989; doi:10.1038/s44161-022-00065-1)
Supplement: Supplementary file 2 — Reporting Summary [file 44161_2022_65_MOESM2_ESM.pdf]

nature portfolio

Corresponding author(s): Miguel Torres

Last updated by author(s): Apr 5, 2022

Reporting Summary

Nature Portfolio wishes to improve the reproducibility of the work that we publish. This form provides structure for consistency and transparency in reporting. For further information on Nature Portfolio policies, see our Editorial Policies and the Editorial Policy Checklist.

Statistics

For all statistical analyses, confirm that the following items are present in the figure legend, table legend, main text, or Methods section.

n/a	Confirmed	
☐	☒	The exact sample size (*n*) for each experimental group/condition, given as a discrete number and unit of measurement
☐	☒	A statement on whether measurements were taken from distinct samples or whether the same sample was measured repeatedly
☐	☒	The statistical test(s) used AND whether they are one- or two-sided<br>*Only common tests should be described solely by name; describe more complex techniques in the Methods section.*
☒	☐	A description of all covariates tested
☐	☒	A description of any assumptions or corrections, such as tests of normality and adjustment for multiple comparisons
☐	☒	A full description of the statistical parameters including central tendency (e.g. means) or other basic estimates (e.g. regression coefficient) AND variation (e.g. standard deviation) or associated estimates of uncertainty (e.g. confidence intervals)
☐	☒	For null hypothesis testing, the test statistic (e.g. *F*, *t*, *r*) with confidence intervals, effect sizes, degrees of freedom and *P* value noted<br>*Give P values as exact values whenever suitable.*
☒	☐	For Bayesian analysis, information on the choice of priors and Markov chain Monte Carlo settings
☒	☐	For hierarchical and complex designs, identification of the appropriate level for tests and full reporting of outcomes
☒	☐	Estimates of effect sizes (e.g. Cohen's *d*, Pearson's *r*), indicating how they were calculated

Our web collection on statistics for biologists contains articles on many of the points above.

Software and code

Policy information about availability of computer code

Data collection	Leica LAS X 3.5.2.18963 software was used to stitch tile-scan stacks. Huygens Professional version 19.10 was used to deconvolve raw images.
Data analysis	Graphpad Prism 8.0.1 was used for data representation and statistical analysis. ITK-SNAP 3.8.0 was used for image segmentation. ImageJ 1.53f was used for image visualization and processing. Paraview 5.4 was used for mesh visualization. NiBabel 3.1.1 was used for reading and writing Nifti image format (segmentation images). All the image processing steps have been implemented in Python 3.8. Scikit-image 0.16.2 was used for denoising and filtering images. Trimesh 3.9.19 and Meshlab 2020.12 were used for mesh analysis and processing. PyMCubes 0.1.2 was used to reconstruct in 3D the segmentation images. The library "Manifold Approximation of 3D Medial Axis" (Shin Yoshizawa, 2011 release) was used to skeletonize the meshes and produce the mid-surfaces. Imaris 9.5.1 has been used by S.M.M. lab to measure the h/w ratios. A new software package for Intersurface maps computation is provided through the open repository Zenodo.

For manuscripts utilizing custom algorithms or software that are central to the research but not yet described in published literature, software must be made available to editors and reviewers. We strongly encourage code deposition in a community repository (e.g. GitHub). See the Nature Portfolio guidelines for submitting code & software for further information.

Data

Policy information about availability of data

All manuscripts must include a data availability statement. This statement should provide the following information, where applicable:

- Accession codes, unique identifiers, or web links for publicly available datasets
- A description of any restrictions on data availability
- For clinical datasets or third party data, please ensure that the statement adheres to our policy

DATA AVAILABILITY

Datasets with the original segmentations, the processed 3D models and the 3D+t models are available at Mendeley Data: https://data.mendeley.com/datasets/t828xhg66k/1 (temporary access at: https://data.mendeley.com/datasets/t828xhg66k/draft?a=33d4dd5d-d49c-4929-b2d5-b41b37d5d418). We recommend opening with the open-source software "Paraview" (www.paraview.org). Instructions for visualizing the 3D images and Videos in ParaView with custom selection of tissues, colouring and viewpoint are provided with these datasets.
Datasets with the source confocal images and original segmentations are available at the IDR repository: https://doi.org/10.17867/10000174

CODE AVAILABILITY
The software package including SurfaceMapComputation and ViewMap is available at Zenodo repository:
https://doi.org/10.5281/zenodo.6390818

Field-specific reporting

Please select the one below that is the best fit for your research. If you are not sure, read the appropriate sections before making your selection.

☒ Life sciences ☐ Behavioural & social sciences ☐ Ecological, evolutionary & environmental sciences

For a reference copy of the document with all sections, see nature.com/documents/nr-reporting-summary-flat.pdf

Life sciences study design

All studies must disclose on these points even when the disclosure is negative.

Sample size	The main sample collection (52 specimens) was used to generate a sequence of developmental stages. The aim was to define a specimen collection with sufficient temporal density to describe the morphological evolution of tissues. In this sense, the determination of the number of specimens is arbitrary. We chose a temporal density within the range of previous developmental Biology studies aiming to generate similar data for other developing organs. The study that has used a temporally denser collection of specimens for mouse development is that described in Musy et al. (Development (2018) 145 (7): dev154856). In that study, they collected embryos every 8 minutes on average. Their study then concluded that it was not possible to detect developmental differences below 2h of difference between embryos. We therefore set our temporal density to 1 specimen every 20 minutes, which would provide around 6 embryos each 2 hours. This was determined as the best compromise between number of specimens and statistical description of morphological trajectories of the developing heart
Data exclusions	No data were excluded
Replication	A second collection was analyzed containing 39 specimens, including controls and mutants. The sample size was estimated from the data obtained with the main collection. This second collection (replication) validated the staging system defined by the first collection. No other hypotheses were tested and only this replication was performed.
Randomization	We did not compare groups of specimens under different treatments or conditions, so randomization is not applicable to any of our studies.
Blinding	Groups were defined by genotype and data acquisition was not blinded regarding group allocation. Nonetheless, for comparison between groups, data were extracted automatically from the 3D images, so no subjective judgment was involved at any step

Reporting for specific materials, systems and methods

We require information from authors about some types of materials, experimental systems and methods used in many studies. Here, indicate whether each material, system or method listed is relevant to your study. If you are not sure if a list item applies to your research, read the appropriate section before selecting a response.

Materials & experimental systems

n/a	Involved in the study	
☒	☐	Antibodies
☒	☐	Eukaryotic cell lines
☒	☐	Palaeontology and archaeology
☐	☒	Animals and other organisms
☒	☐	Human research participants
☒	☐	Clinical data
☒	☐	Dual use research of concern

Methods

n/a	Involved in the study	
☒	☐	ChIP-seq
☒	☐	Flow cytometry
☒	☐	MRI-based neuroimaging

Animals and other organisms

Policy information about studies involving animals; ARRIVE guidelines recommended for reporting animal research

Laboratory animals	A total of 91 laboratory mouse (Mus musculus) embryos between days 7 and 8.5 of gestation were used. This corresponded to 52 embryos with fluorescent reporter transgenes and Cre lines: Mesp1-Cre, ROSA26R-mTmG and ROSA26R-Tomato in the M.T. laboratory and 39 unlabeled embryos in the S.M.M. laboratory. A total of 4 males and 10 females females were used as progenitors

	for embryo collection. Mouse alleles used in the manuscript are listed including bibliographic references and allele identities at the 'Mouse Genome Informatics' data base (MGI, http://www.informatics.jax.org/). Mesp1cre (MGI:2176467), Rosa26RmTmG (MGI:3716464), ROSA26RTomato (MGI:6260212), C57BL/6 (Charles River), Nodalflox (MGI:3056345), Nodalnul , Hoxb1Cre (MGI:2668513). Adult mice were housed in an air-conditioned room with a 12h light/dark cycle and free access to water and chow diet. Embryos imaged to generate the MT collection were maintained on a mixed 129/SvJ x CD1 genetic background. Embryos imaged to generate the SMM collection were maintained on a mixed C57BL/6 x 129/SvJ genetic background. All animal procedures in the MT laboratory were approved by the CNIC Animal Experimentation Ethics Committee, by the Community of Madrid (Ref. PROEX 144.1/21) and conformed to EU Directive 2010/63EU and Recommendation 2007/526/EC regarding the protection of animals used for experimental and other scientific purposes, enforced in Spanish law under Real Decreto 1201/2005. Nodal mutants were housed in the Laboratory of Animal Experimentation and Transgenesis of the SFR Necker, Imagine Campus, Paris. Animal procedures were approved by the ethical committees of the Université de Paris and by the French Ministry of Research.
Wild animals	The study does not include wild animals
Field-collected samples	The study does not include field-collected samples
Ethics oversight	All animal procedures in the MT laboratory were approved by the CNIC Animal Experimentation Ethics Committee and by the Community of Madrid (Ref. PROEX 144.1/21) and conformed to EU Directive 2010/63EU and Recommendation 2007/526/EC regarding the protection of animals used for experimental and other scientific purposes, enforced in Spanish law under Real Decreto 1201/2005. Nodal mutants were housed in the Laboratory of Animal Experimentation and Transgenesis of the SFR Necker, Imagine Campus, Paris, and animal procedures were approved by the ethical committees of the Université de Paris and by the French Ministry of Research.

Note that full information on the approval of the study protocol must also be provided in the manuscript.
